# Machine Learning Analysis of τRAMD Trajectories to Decipher Molecular Determinants of Drug-Target Residence Times

**DOI:** 10.3389/fmolb.2019.00036

**Published:** 2019-05-24

**Authors:** Daria B. Kokh, Tom Kaufmann, Bastian Kister, Rebecca C. Wade

**Affiliations:** ^1^Molecular and Cellular Modeling Group, Heidelberg Institute for Theoretical Studies (HITS), Heidelberg, Germany; ^2^Department of Biosciences, Heidelberg University, Heidelberg, Germany; ^3^Zentrum für Molekulare Biologie der Universität Heidelberg, DKFZ-ZMBH Alliance, Heidelberg University, Heidelberg, Germany; ^4^Interdisciplinary Center for Scientific Computing (IWR), Heidelberg University, Heidelberg, Germany; ^5^Department of Physics, Heidelberg University, Heidelberg, Germany

**Keywords:** drug-protein residence time, machine learning, drug-target binding kinetics, structure-kinetic relationships (SKRs), heat shock protein 90 (HSP90), molecular dynamics simulation, tauRAMD

## Abstract

Drug-target residence times can impact drug efficacy and safety, and are therefore increasingly being considered during lead optimization. For this purpose, computational methods to predict residence times, τ, for drug-like compounds and to derive structure-kinetic relationships are desirable. A challenge for approaches based on molecular dynamics (MD) simulation is the fact that drug residence times are typically orders of magnitude longer than computationally feasible simulation times. Therefore, enhanced sampling methods are required. We recently reported one such approach: the τRAMD procedure for estimating relative residence times by performing a large number of random acceleration MD (RAMD) simulations in which ligand dissociation occurs in times of about a nanosecond due to the application of an additional randomly oriented force to the ligand. The length of the RAMD simulations is used to deduce τ. The RAMD simulations also provide information on ligand egress pathways and dissociation mechanisms. Here, we describe a machine learning approach to systematically analyze protein-ligand binding contacts in the RAMD trajectories in order to derive regression models for estimating τ and to decipher the molecular features leading to longer τ values. We demonstrate that the regression models built on the protein-ligand interaction fingerprints of the dissociation trajectories result in robust estimates of τ for a set of 94 drug-like inhibitors of heat shock protein 90 (HSP90), even for the compounds for which the length of the RAMD trajectories does not provide a good estimation of τ. Thus, we find that machine learning helps to overcome inaccuracies in the modeling of protein-ligand complexes due to incomplete sampling or force field deficiencies. Moreover, the approach facilitates the identification of features important for residence time. In particular, we observed that interactions of the ligand with the sidechain of F138, which is located on the border between the ATP binding pocket and a hydrophobic transient sub-pocket, play a key role in slowing compound dissociation. We expect that the combination of the τRAMD simulation procedure with machine learning analysis will be generally applicable as an aid to target-based lead optimization.

## Introduction

The binding affinity of small compounds to their target is commonly used as a selection criterion in drug design pipelines, both for the early screening of chemical libraries and for the subsequent lead optimization. Recent studies have, however, shown that drug efficacy often correlates better with the residence time than with the binding affinity of drugs (Copeland et al., 2006; Schuetz et al., 2017). These observations suggest that the optimization of the kinetic properties of drug candidates at an early stage of the drug design process would be advantageous.

The computation of drug-target binding kinetics by using MD simulations is more challenging than the computation of binding affinity (Romanowska et al., 2015). A major problem in using conventional MD simulations for computing binding kinetic parameters is the need to sample the intermediate transition states between the bound and unbound states, which is not required for the calculation of binding affinity. This poses tremendous challenges for brute-force conventional MD sampling, whose application is so far limited to computation of the binding kinetics of small molecules to small proteins, e.g., benzamidine to trypsin, which still requires extensive millisecond simulations (Dror et al., 2011; Wu et al., 2016). Reconstruction of a single dissociation event for a pharmacologically relevant compound, which typically occurs on the time-scale of minutes or hours, is currently not feasible from conventional MD simulations. To overcome this limitation, a range of enhanced sampling techniques has been explored recently (Bruce et al., 2018). Some of them are aimed at the reduction of the configurational space to be sampled for the computation of binding kinetic rates, e.g., metadynamics (Tiwary et al., 2015, 2017), weighted ensemble methods (Dickson and Lotz, 2016; Dixon et al., 2018), or milestoning (Tang and Chang, 2017) [a detailed review can be found elsewhere (Mollica et al., 2016; Dickson et al., 2017)]. Although these methods are designed for the prediction of the absolute values of binding and unbinding rates within a reasonable computation time, they are still very computationally demanding and require high user expertise, which impedes the implementation of these methods in drug design pipelines. Furthermore, in addition to the limitations arising from the selection of the sub-space to be sampled, intrinsic limitations of the underlying physical model of molecular interactions, such as the force field and the water model, may affect the accuracy of the computed rates.

While absolute values are difficult to attain, it has been demonstrated recently that the relative values of unbinding rates for a series of ligands of a particular target are more robust to these limitations (Marques et al., 2019). In line with this finding, computationally efficient approaches that provide estimates of the relative residence times for a set of compounds have been reported. Instead of deriving the residence time from the energetic profile of dissociation paths, these techniques allow estimation of relative τ values from the times required for ligand egress during enhanced sampling simulations. The residence times obtained can then be scaled for direct comparison with experimental data. One example of this approach is scaled MD (Mollica et al., 2015; Schuetz et al., 2018a) in which the potential of the system is rescaled during simulations. Another approach, recently developed in our group, is the τRAMD method (Kokh et al., 2018), which employs multiple short random acceleration MD, RAMD, simulations to generate ligand dissociation trajectories. Relative drug-protein residence times are estimated from the times required for the ligand to leave the binding pocket in simulations started from the structures of protein-ligand complexes. In RAMD (Lüdemann et al., 2000), an additional randomly oriented force is applied to the ligand's center of mass and its direction is altered during the simulations, depending on the motion of the ligand. RAMD was originally developed to explore ligand egress routes from protein binding sites [see e.g., (Winn et al., 2002; Schleinkofer et al., 2005)], where simulated trajectories were employed to explore ligand unbinding pathways and mechanisms. In the τRAMD procedure, many trajectories are generated (usually more than 40 for each compound) and each trajectory contains hundreds of thousands of snapshots that may contain important information for the ligand unbinding rate. The value of extracting molecular features from MD simulations as fingerprints for building machine learning (ML) models to predict molecular properties has been demonstrated in Re. (Riniker, 2017). Here, we explore whether fingerprint-based ML techniques can aid the detection of features important for drug-target residence time in RAMD trajectories and, furthermore, improve the robustness of the estimated residence times.

ML has been applied for drug-target τ prediction in several studies. Qu et al. (2016) derived quantitative structure-kinetics relationships (QSKRs) for a set of HIV-1 protease inhibitors by using Volsurf descriptors. Chiu and Xie (2016) went beyond a static model by accounting for flexibility with a coarse-grained normal mode analysis to classify HIV-1 protease inhibitors in binding kinetics classes using a multi-target ML approach. Comparative Binding Energy (COMBINE) analysis (Ortiz et al., 1995; Perez et al., 1998), in which PLS (Partial Linear Regression Projection to Latent Structures) is used to reweight components of the bound protein-ligand interaction energies to predict binding properties, has recently been applied to datasets of HSP90 and HIV-1 protease inhibitors (Ganotra and Wade, 2018) and was found to give models with good predictive ability for residence time. It should be noted that the COMBINE analysis method was originally developed for the prediction of binding affinity for congeneric series of compounds. While compounds with a common scaffold are required for good prediction of the equilibrium dissociation constant, K_D_, a good prediction of the off-rate could be obtained for a dataset of diverse compounds from analysis of the bound protein-ligand complexes (Ganotra and Wade, 2018) suggesting that differences in the unbound state are less important for off-rate than for binding affinity. Huang et al. (2019) applied PLS analysis to interaction-energy fingerprints extracted from snapshots of steered MD ligand dissociation trajectories to obtain a predictive model for residence time for a set of HIV-1 protease inhibitors and found that important interactions for determining τ were in the first half of the dissociation processes. This is consistent with a previous steered MD study of HIV-1 protease inhibitor dissociation in which the strength of the ligand-protein hydrogen bond network of the bound state was found to be crucial for the dissociation process (Li et al., 2011), as well as with the above-mentioned models based solely on analysis of the bound state.

In the present study, we use our previously published τRAMD simulation results for a data set of 70 inhibitors of the cancer target HSP90 for which off-rates were measured by surface plasmon resonance (SPR) (Amaral et al., 2017; Kokh et al., 2018). These compounds bind in the ATP binding site of the N-terminal domain of human HSP90 (N-HSP90α, residues 9-236; NP_005339). The τRAMD procedure gave predictions of relative residence times with an accuracy of about 2.3τ for 78% of the compounds and < 2.0τ within congeneric series. It was found that the computed residence times were sensitive to the quality of the underlying MD simulations of the protein-ligand complexes. For some compounds, deficiencies in the force field or inaccuracies in the docking pose led to notable underestimation of the residence time, although within a series of compounds with the same binding scaffold and small fragment substitutions, the ranking of the residence time was well-reproduced. The latter result suggests that the inaccuracy of the simulations of the bound state may be overcome in τRAMD simulations if the transition state is the main determinant of the variation in residence time within a congeneric series of compounds.

Here, we have performed τRAMD simulations for an additional 25 HSP90 inhibitors, whose binding kinetics were recently reported (Schuetz et al., 2018b). We have then combined these simulations with our previous simulations (Kokh et al., 2018), and applied ML approaches to the combined dataset of simulated trajectories for 94 HSP90 inhibitors.

**Figure 1:**
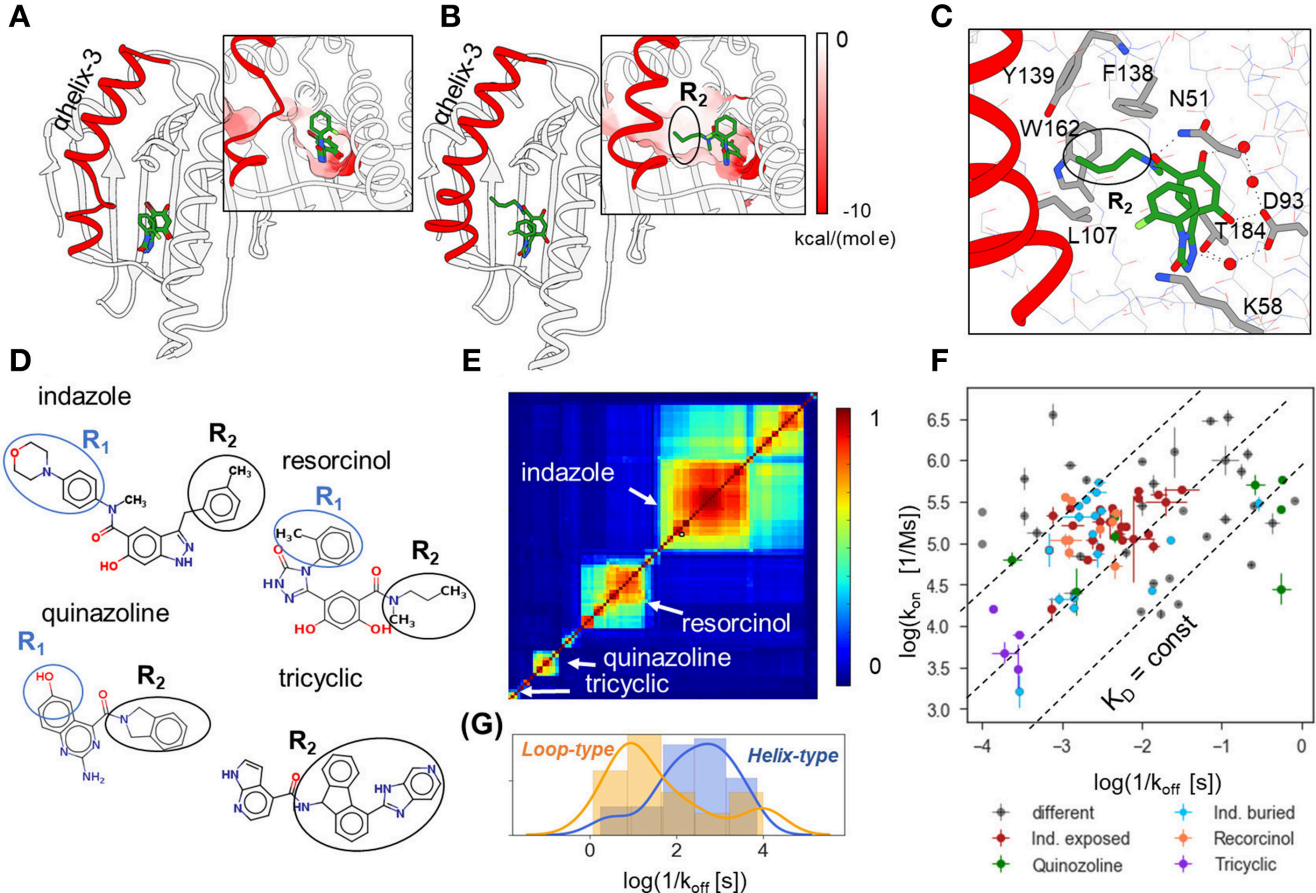
Structural and binding kinetic properties of the dataset of 94 N-HSP90 inhibitors. **(A,B)** Two conformations of the ATP binding site in N-HSP90 with a bound ligand shown in stick representation with coloring by atom type; α-helix3 (highlighted in red) can be distorted in the middle (loop-type conformation **(A)**, compound **5** PDB ID 5J2X) or complete (helix-type conformation **(B)**, compound **13**, PDB ID 5J9X) (Amaral et al., 2017); the molecular surface of the binding pocket colored by the Coulomb potential is shown in insets for both conformations: the ATP binding site has predominantly negative charge (red), whereas the transient sub-pocket under α-helix3 is mostly hydrophobic. **(C)** Protein-ligand contacts for helix-binding compounds are illustrated for compound **13**, (PDB ID 5J9X): the ligand-protein binding network consisting of D93, T184, and three water molecules (red spheres) is common to all compounds; compounds bound to the helix-conformation of the binding site also interact with F138 and may interact with residues in the hydrophobic pocket, such as W162 and Y139. **(D)** 2D representation showing the four main groups of compounds discussed in the text. **(E)** Similarity matrix of the 90 N-HSP90 inhibitors generated using Maestro [(Schrödinger, 2019); see text]. **(F)** Distribution of the experimental binding rate constants of the entire set of compounds. The three largest groups of compounds are colored as denoted in the legend: “Ind. exposed”—indazole-based compounds with different R_1_ fragments, “Ind. buried”—indazole compounds with different R_2_ fragments, compounds with resorcinol and quinazoline scaffolds, as well as bulky compounds with a tricyclic fragment and different ATP-pocket binding core. **(G)** Distribution of residence times of the helix-binding and loop-binding compounds.

N-HSP90 is a challenging target for the prediction of binding kinetics, as it has a flexible ATP binding site lined by the unstable α-helix3 that can adopt either “helical” or “loop” conformations (see Figures 1A,B), depending on the ligand bound. The “helical” conformation contains an additional hydrophobic sub-pocket adjacent to the ATP binding site, which provides space for substitutions on ‘helix-binders’ (fragment R_2_, see Figures 1C,D), while this fragment is absent in the compounds bound to the “loop” conformation (‘loop-binders’). It has been recently demonstrated that the binding kinetics of resorcinol inhibitors of HSP90 is related to the protein binding site conformation in the bound complex, and that the R_2_ substitution can effectively stabilize α-helix3 and result in lower binding and unbinding rates for ligands with such fragments (Amaral et al., 2017). In particular, ligands with large R_2_ substitutions, such as tricyclic compounds (Figure 1D), generally have the slowest binding and unbinding kinetics (Figure 1F).

The set of 94 compounds considered in the present study contains molecules with 11 different scaffolds: resorcinol (28), hydroxyindazole (47), benzamide (3), aminoquinazoline (8), aminopyrrolopyrimidine (2), 7-azaindole (2), aminothienopyridine (1), imidazopyridine (1), 6-hydroxyindole (1), and adenine (1) (with the number of compounds given in brackets; see Supplementary Tables 1 and 2; SMILES of all studied compounds are given in Supplementary Table 4). The scaffold occupies the ATP binding pocket and binds to D93 as illustrated in Figure 1C for an indazole-based compound. The three most populated scaffolds are shown in Figure 1D, along with an example of compounds with different binding scaffolds but a common tricyclic group, which will be discussed below. Further, the resorcinol compounds with triazole and 2-methylbenzyl solvent-exposed groups and different buried fragments, illustrated in Figure 1D, build a sub-group of 8 compounds. Following Schuetz et al. (2018b), one can also distinguish two sub-groups of indazole compounds: (i) indazole-exposed: 24 compounds with a 3-methylbenzyl R_2_ moiety in the hydrophobic sub-pocket and different exposed R_1_ fragments, and (ii) indazole-buried: 17 compounds with an exposed 4-(4-morpholinyl) phenyl R_1_ fragment and different buried R_2_ fragments (see Figure 1D). The rest of the compounds is quite diverse, as can be seen from the 2D similarity plot generated using Maestro software (Schrödinger, 2019) by hierarchical clustering of compounds based on their 2D fingerprint similarity in Figure 1E. There are both loop- and helix-binders of different scaffolds, though the sub-set of loop-binders is much smaller (only 13) than the helix-binders.

The experimental binding kinetics data for the full compound set (Amaral et al., 2017; Kokh et al., 2018; Schuetz et al., 2018b) are plotted in Figure 1F. Both off-rates (k_off_ = 1/τ) and on rates (k_on_) vary by several orders of magnitude and there is no clear correlation between them, indicating that both the height of the transition barrier and the free energy of the bound state vary across the compound set. Notably, the helix-binders generally have longer residence times than the loop-binding compounds (Figure 1G).

Here, we built ML models based on the τRAMD dissociation trajectories for this data set aimed at: (i) investigating whether residence time can be deduced from the protein-ligand contact occurrence in τRAMD ligand dissociation trajectories, in particular for the cases where the relative residence times derived from the lengths of τRAMD trajectories are consistently underestimated; and (ii) identifying molecular properties that affect ligand residence time and that can be used to guide the design of ligands with altered binding kinetics.

## MethodS and Materials

**Figure 2:**
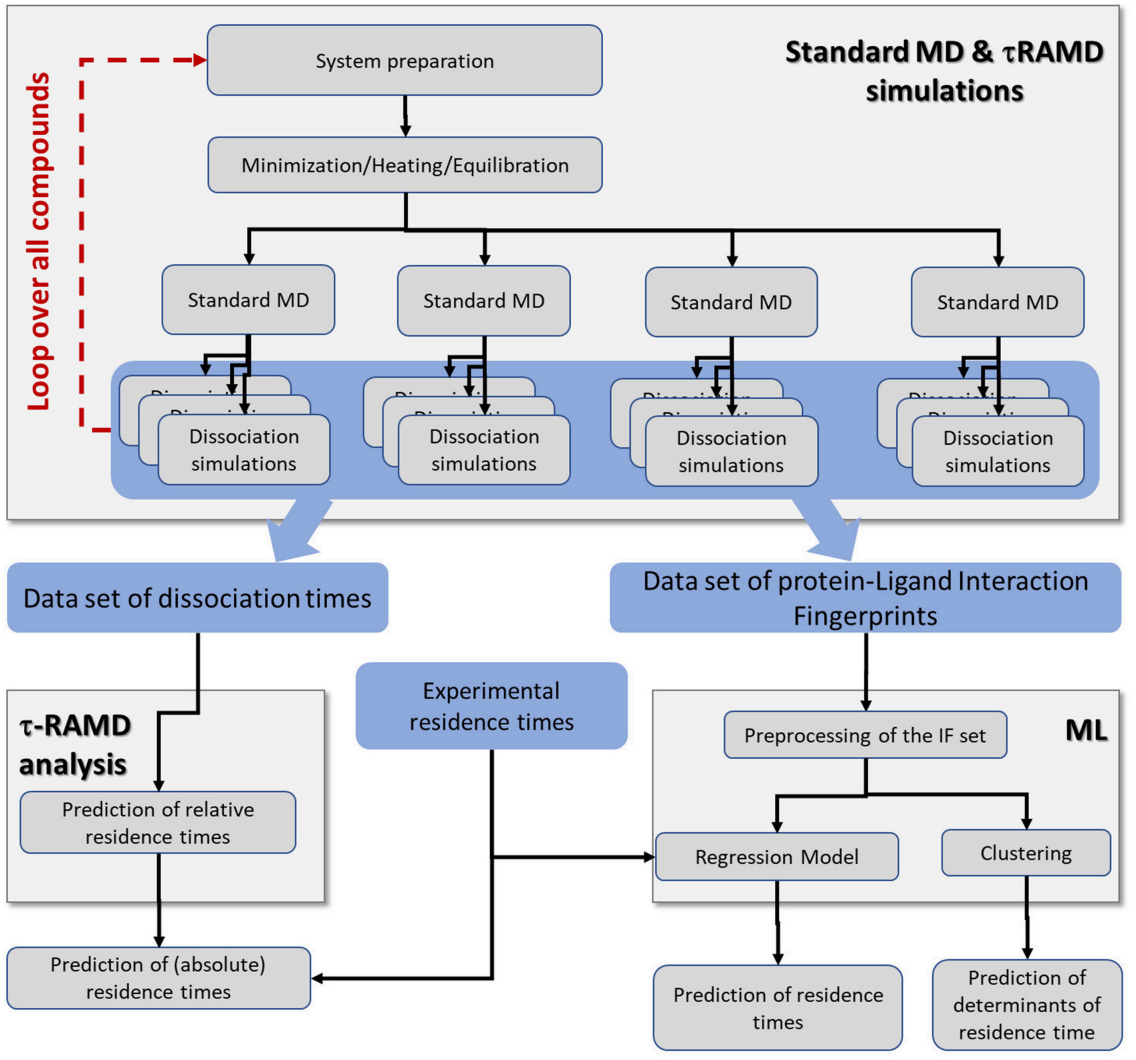
Workflow incorporating the simulation protocol for τRAMD simulations and the ML analysis. The τRAMD simulations provide (i) computed relative residence times, and (ii) trajectories that are used for analysis of protein-ligand contacts and building a ML regression model for prediction of residence times and determining the factors governing residence time (see section Methods and Materials); data sets generated and elements of simulation workflow are highlighted by blue and gray background, respectively.

An overview of the simulation workflow is given in Figure 2. For each compound, the τRAMD procedure was performed, which consists of the preparation of the solvated protein-ligand complex, the equilibration of the system using multiple replicas of standard MD simulation, and then the simulation of multiple RAMD ligand dissociation trajectories. The τRAMD relative residence times are obtained using the protocol reported by Kokh et al. (2018). In the second part of the workflow, the protein-ligand contacts (referred to hereafter as interaction fingerprints, IFs) are extracted from τRAMD dissociation trajectories. Then, for all compounds, the IFs are transformed into a set of features for the ML analysis, which includes the clustering of the ligand dissociation properties and the building of regression models for residence time based on available experimental binding kinetics data (see the next section). The workflow is described in detail in the following sections.

### Kinetic and Structural Data for the Dataset of HSP90 Inhibitors

We employed 69 of the 70 compounds with structural and kinetic data in Kokh et al. (2018). One compound [**70** in Kokh et al. (2018)] was eliminated from the dataset because its complex with N-HSP90 was structurally unstable during MD equilibration. For two compounds with affinities and long residence times beyond the measurement range (PDB ID 2VCI and 5NYI, compounds **1** and **4**, see Supplementary Tables 1, 2), we used the lower limit values of k_off_ = 10^−4^ s^−1^ and K_D_ = 10^−9^ M^−1^. Additionally, we studied 25 compounds from Schuetz et al. (2018b). Since there are no crystal structures of protein-ligand complexes available for these 25 compounds yet, the ligands were modeled in the N-HSP90 binding site using (MOE., 2017) on the basis of similarity to available crystal structures for similar compounds: PDB ID 5OCI and 6EFU for the indazole compounds, and PDB ID 5J86 for the resorcinol compounds.

### MD and RAMD Simulations

The τRAMD protocol as described by Kokh et al. (2018) was followed. Here, we outline this protocol briefly for completeness. First, the starting structure of each protein-ligand complex was protonated at pH 7. The ligand was protonated using MOE (MOE., 2017) and the protein was protonated using PDB2PQR (Unni et al., 2011). The atomic partial charges of the ligands were assigned using the RESP approach (Bayly et al., 1993) with the molecular electrostatic potential computed using *ab initio* quantum mechanical calculations performed at the HF level with a 6-31G^*^(1d) basis set using the Gamess software (Gordon and Schmidt, 2005). The protonated protein-ligand complex was solvated in a periodic box of TIP3P water molecules and Na^+^ and Cl^−^ ions at an ionic strength of about 150 mM. Crystallographic water molecules were retained. The system was energy minimized, gradually heated and shortly equilibrated with gradually decreasing restraints on all non-hydrogen atoms of the protein, ligand, and crystallographic water molecules using the AMBER molecular dynamics simulation software (Case et al., 2016). Simulations were run under NPT conditions (Langevin thermostat and barostat). Then the coordinates of the preliminary equilibrated binding complex were transferred to the NAMD program (Phillips et al., 2005) and used as the input for heating and equilibrating the system. The coordinates and velocities obtained after 30–40 ns of equilibration were used to initiate simulations of ligand dissociation using the RAMD method with a randomly oriented force on the ligand with a constant magnitude of 14 kcalmol^−1^Å^−1^. Every 100 fs, the orientation of the force was randomly re-initialized if the center of mass of the ligand had moved < 0.025Å. The simulations were stopped when the center of mass of the ligand had moved 30 Å from the original bound position.

At least four MD equilibration replicas were prepared and from each replica 10–20 RAMD dissociation trajectories were generated. The relative residence time was defined as the time when a dissociation event was observed in 50% of the trajectories. It was computed for each starting replica and then averaged over all replicas simulated. Sufficient sampling to compute residence time was ensured by increasing the number of equilibration replicas and/or the number of dissociation trajectories if necessary as discussed in Kokh (2018).

### Feature Generation

**Figure 3:**
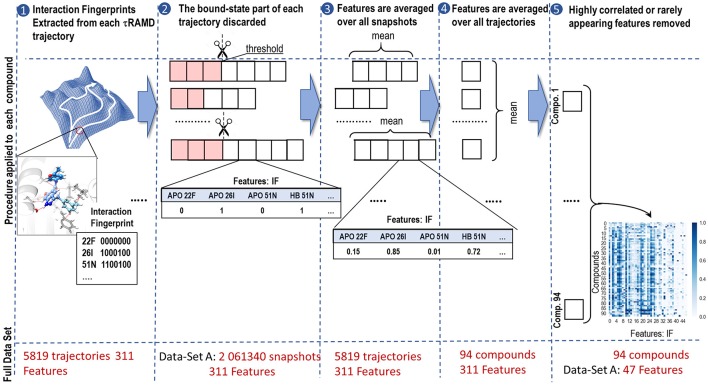
Workflow illustrating the generation of features from simulated τRAMD trajectories: (1) Extraction of interaction fingerprints as features for ML; (2) Discarding of the bound state part of the trajectory (highlighted in pink). The discarded part of the trajectory depends on the threshold used, resulting in data-sets A, B, and C (see text for details); (3) Averaging of the features over all snapshots in each trajectory; (4) Averaging of the features over all trajectories for each compound; (5) Removal of features that appear rarely or are strongly correlated with other features. The size of each set of data at each stage of the workflow is indicated in red.

The feature generation procedure is illustrated in Figure 3. First, a set of interaction fingerprints (IF) was obtained from the τRAMD dissociation trajectories (40–100 trajectories for each compound) using the following protocol: (i) the position of the center of mass of the ligand and the coordinates of the protein and the ligand atoms were extracted from each trajectory frame and stored using a tcl script for the VMD program (Humphrey et al., 1996) (snapshots illustrating egress routes and residues contacting the ligand during dissociation are visualized in Supplementary Figure 1); (ii) the coordinates extracted in (i) were used to generate interaction fingerprints for each frame using an OpenEye's OEChem Toolkit (OpenEye., 2018) as 7-bit strings encoding hydrophobic, aromatic face-to-face and edge-to-face, H-bond donor/acceptor and cationic/anionic interaction types (Marcou and Rognan, 2007; Mysinger et al., 2012). Then the interaction fingerprints were grouped into four categories of protein-ligand contacts: hydrogen-bond (HB), aromatic (ARO), ionic (IP), and apolar (APO) interactions, and each category was assigned a value of 1 or 0 according to whether the contact type was, respectively, present or not; (iii) finally, the bound-state part of the trajectory was removed and only the part of the trajectory covering the transition of the ligand from the bound to the unbound state was used for further analysis (step 2 in Figure 3). Since the threshold for the separation between the bound- and transition parts can be defined arbitrarily, we explored three possible threshold definitions (these will be referred to as data sets, A, B and C, hereafter): (A) when two IF observed in the bound state (i.e., in the first frame of a trajectory) are lost, or (B) when 20%, or (C) when 60% of the bound-state contacts are lost (the size of each data set is given in Supplementary Table 3).

Although the sequence of interaction events may bear important information about the ligand dissociation mechanism, preliminary tests showed that the RAMD trajectories generated did not permit us to build a reliable time-dependent model, probably due to having insufficient number of snapshots along the ligand dissociation trajectories as the artificial random force accelerated dissociation. Therefore, we eliminated time dependence in our data by computing the occurrence of each type of contact in each trajectory and averaging them over all trajectories for a particular compound (steps 3 and 4 in Figure 3). This provided us with a matrix of 94 labels (compounds) x 311 features (fingerprints). This matrix was further reduced by partial elimination of the noise in the data set. In particular, since we did not expect that a very rare contact would affect dissociation rate, we excluded features that were found in fewer than 5% of the frames for any compound. This reduced the number of features to 68/69/75 for the complete A/B/C data-sets, respectively. Then, we performed preliminary correlation analysis and removed one of the features from each pair that had a correlation R^2^ > 0.9, thus further reducing the number of features to 47/48/57 for the data-sets A/B/C, respectively (see Supplementary Table 3).

To explore the influence of molecular properties on the residence time, we additionally generated a set of molecular features, MFs, for all compounds using MOE (MOE., 2017). The MFs include the number of bonds of different types, the number of atoms with hydrogen-bond properties, the number of heavy atoms, and the solvation energy (the complete list is given in Supplementary Table 2). For testing the importance of these molecular features, they were either added to the IFs of data-set A or used as a separate feature set.

### Machine Learning Protocol

The scikit-learn Python library (Pedregosa et al., 2011) was used for all machine learning (ML) procedures.

#### Regression Analysis

The data sets were normalized by transforming each feature vector to the interval [0:1]. The ML models were trained and tested against measured log(1/k_off_) values. Two regression models (RM), one linear—Ridge Linear Regression with L^2^ regularization terms (LR)—and one non-linear—Support Vector Regression (SVR)—were found to be more balanced and slightly more stable in cross-validation than the other methods tested (Partial Least Squares, Random Forest and Gaussian Boosting Regression). Additionally, a dummy regression model with the mean value of the training set as a null-hypothesis (referred to as Dummy Regressor hereafter) was used as a control.

The modeling workflow consisted of the following steps (as illustrated in Supplementary Figure 2):

- **Split the data set into a training (internal) set and an external test set**. For the test set, we selected 20% of compounds from the data set while ensuring that the test set contained 2 randomly selected compounds from the outlier subset of 8 quinazolines (compounds **58–65**) and six other compounds (**11**, **17**, **30**, **66**, **67**, **69**) as defined in Kokh et al. (2018); these compounds are highlighted in yellow in Supplementary Table 2), and 20% (i.e., at least 9 compounds) from the subset of indazole compounds (compound scaffolds are given in Supplementary Table 2). The rest of the test set was selected randomly from the remaining compounds. The purpose of this selection was two-fold: (1) to test the prediction accuracy for compounds that were considered as outliers in τRAMD simulations; and (2) to avoid over-representation of the indazole compounds in the training set, since they constitute almost 50% of all compounds in the data set.
- **Selection of hyperparameters for the two regression models, LR and SVR** (this block is zoomed in in Supplementary Figure 2). The internal training set was used for the selection of hyperparameters. The following parameters were optimized: coefficient of the regularization term for the LR model; kernel coefficient (the RBF kernel was used), parameter of the loss function, and coefficient of the error term for the SVR model. We employed exhaustive grid-search with 10-fold cross-validation (using random permutation splitting with a validation test set size of 20%). The results of the optimization procedure are given in Supplementary Data and illustrated in Supplementary Figures 3, 4.
- **Training and testing of the models**. After the hyperparameters were selected, 10 cross-validation runs were performed on the internal training set. In each round, two regression models, LR and SVR, were trained on a sub-set of the internal training set and then the mean absolute error, MAE, and the ${\text{Q}}_{\text{F}3}^{2}$ metric, reported as the most reliable metric for the evaluation of the regression models (Todeschini et al., 2016), were computed for the training and validation sub-sets (generated using random permutation splitting with a validation sub-set size of 20%), as well as for the external test set (all for the residence time on a log_10_ scale; for more details, see Supplementary Information). Additionally, the same data sub-sets were used to evaluate the Dummy model and the τRAMD simulations.

Then new internal training/external test set combinations were generated step (i) and the steps (ii–iii) were repeated. All MAE and ${\text{Q}}_{\text{F}3}^{2}$ values obtained in these calculations were stored. Altogether, we performed 200 computation rounds, each with a different split of training and test sets, to gain proper statistics. The histograms of the MAE distributions obtained for each ML method were compared with those for the Dummy model for control; histograms of MAE and ${\text{Q}}_{\text{F}3}^{2}$ were compared with the corresponding distributions obtained from the τRAMD protocol. The complete procedure for 100 rounds takes about 1.5 h on a laptop with an Intel Core i5-5200U, 2.2 GHz processor.

#### Clustering

We employed a Gaussian Mixture Model (GMM) for the classification of the compounds by their IFs in the data sets A for all compounds and for the sub-set of indazole-based compounds only. The feature set was normalized by transforming to the interval [0:1], as for the regression models. For the scikit-learn GMM function, we used an option where each component has its own multivariate covariance matrix. To estimate the optimal number of clusters, we used the Akaike information criterion (see Supplementary Information for details). Following a scan of cluster size, 6 clusters were chosen on the basis of minimum loss of information for the complete data set of 94 compounds (A) and 4 clusters for the indazole sub-set of the data set A (Ind) (see Supplementary Figures 5A,B). For each dataset, 50 independent repeats of clustering were performed. For each clustering round, the clusters were ordered by increasing average residence time of the inhibitors belonging to each cluster, and the weights of all features in each cluster were stored. Finally, for each dataset, the mean cluster residence time, τ_c_, over the 50 clusterings was computed for each of the clusters (from their average residence times), with the first having the shortest τ_c._

Further, for the indazole subset (Ind), we explored how some selected structural properties of the compounds are distributed over the clusters. For this, we selected two sets of small fragments that might affect the dissociation rate constant (see Supplementary Figure 6): (i) seven types of solvent-exposed fragments (i.e., different classes of the R_1_ substitution (Figure 1D) and six types of buried fragments (i.e., R_2_, placed in the hydrophobic sub-pocket, see Figure 1C). The number of compounds in each cluster with the corresponding R_1_ and R_2_ fragments was computed and normalized by the cluster size.

## Results and Discussion

### τRAMD Simulations

**Figure 4:**
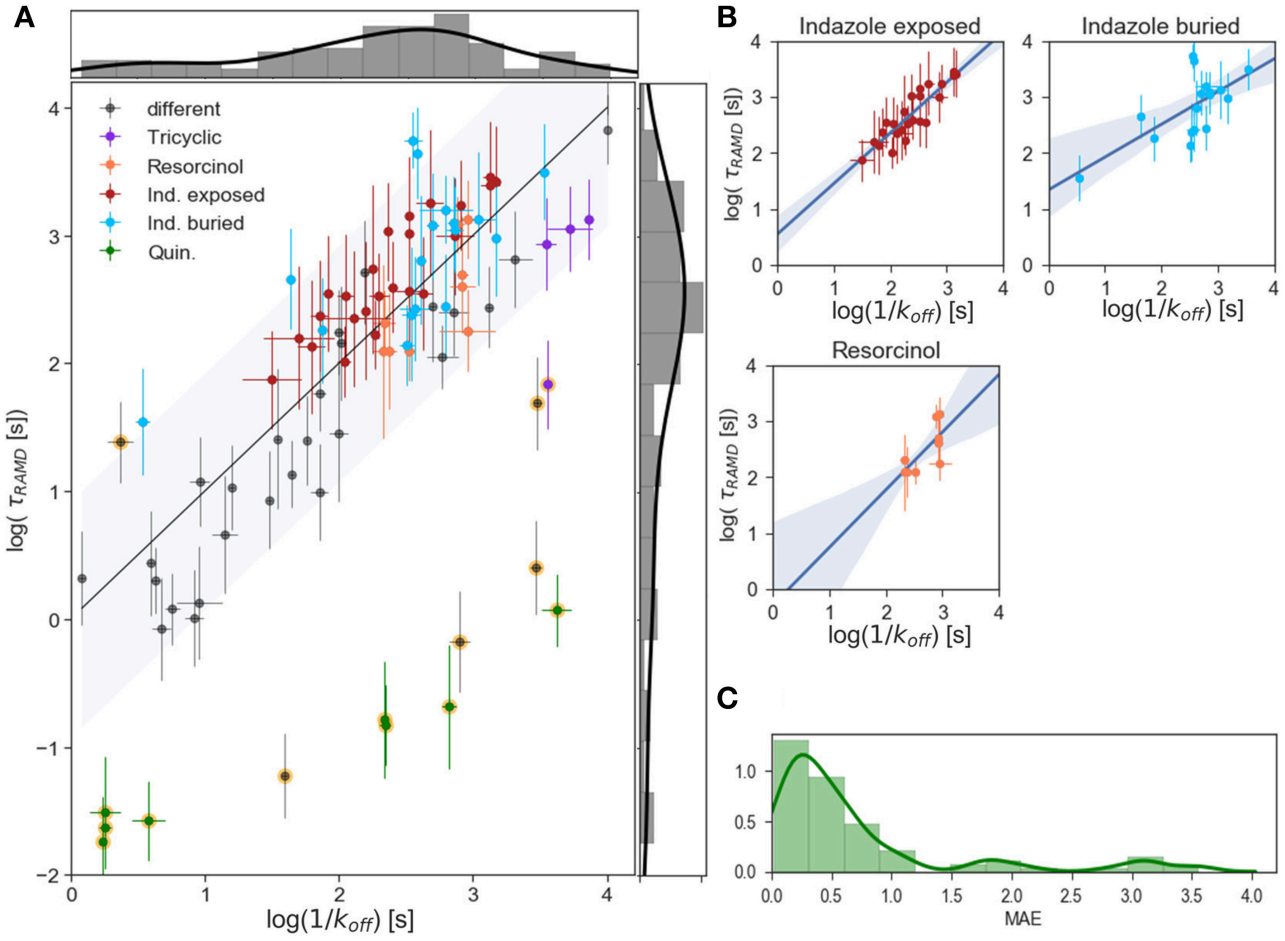
Results of τRAMD simulations. **(A)** Scaled τRAMD residence times plotted vs. measured log (1/k_off_) values on a logarithmic scale for the complete set of compounds. The τRAMD residence times are scaled according to the linear fitting (log(τ_RAMD_) = 0.39*log(1/k_off_) −0.52) of all compounds except for the 13 outliers identified in Kokh et al. (2018) (shown with background yellow circles). Two groups of indazoles (with different exposed R_1_ and buried R_2_ fragments, see Figure 1D), resorcinol and quinazoline compounds, as well as compounds with a tricyclic fragment, are colored as denoted in the legend; other compounds are shown in gray. The black line shows the one-to-one correspondence of the computed and experimental data and the interval within 1.5-fold of the mean of the residuals (0.9 log([s])) is shown by the gray area. The distributions of measured and τRAMD computed residence times are shown along the X and Y axes, respectively. **(B)** Linear fitting with 95% confidence interval for several sub-groups of compounds highlighted in **(A)**: indazole exposed, indazole buried, and resorcinol colored as in **(A)**. **(C)** Histogram showing the distribution of the mean absolute error, MAE, of τRAMD residence times relative to measured values; the long tail arises from the outliers.

Computed relative residence times obtained from the τRAMD simulations for the 94 compounds are shown vs. measured 1/k_off_ values on the logarithmic scale in Figure 4A. As discussed in our previous study (Kokh et al., 2018), 14 compounds from the dataset are outliers: compounds **11**, **17**, **30**, **66**, **67**, **69**, and **8** quinazoline compounds (highlighted in yellow in Figure 4A). Without the outliers, i.e. for 80 compounds (85% of the data set), the correlation coefficient R^2^ = 0.75, MAE = 0.39 ± 0.06, and the mean prediction uncertainty, MPU, is 3.1 τ on average, which is somewhat higher than in the set of 70 compounds studied previously (Kokh et al., 2018) (R^2^ = 0.86 and MPU = 2.3τ for 78% of the compounds, i.e. 55 compounds after omission of outliers).

To understand the reason for this difference, one has to look at the simulation results for the indazole compounds since most of the added compounds are indazoles. 17 out of the 25 additional compounds have an indazole scaffold with a buried 3-methylbenzyl R_2_ substituent and different exposed R_1_ fragments (shown in dark red in Figures 1F, 4A). This group has a computed τ that is systematically longer by approximately 0.5 log units than the value from the linear fit for the other compounds, despite showing a good correlation with the experimental τ values within the group (R^2^ = 0.86, MAE = 0.34, Figure 4B). In contrast, variation of the buried R_2_ fragment in the indazoles leads to a large and non-specific deviation of computed τ values from the fit. Specifically, a series with 4-(4-Morpholinyl) phenyl substitutions in indazole compounds (group colored in cyan in Figures 1F, 4A,B) has a correlation coefficient with experimental data of R^2^ = 0.67, MAE = 0.43. Similarly, a subgroup of 6 resorcinol compounds shown with different R_2_ (shown in Figure 1D, their residence times are colored in orange in Figures 4A,B) substituents has a low correlation, R^2^ = 0.72, MAE = 0.32. The mean prediction uncertainties for the latter three groups are 2.3, 4.3, and 2.2 τ, respectively.

One possible explanation for the poorer correlations for subgroups of compounds with different R_2_ fragments is uncertainty regarding the structure of the bound-state of the protein-ligand complex. All 21 indazole and 6 resorcinol compounds mentioned above were modeled using a template structure since crystal structures were not available for these complexes. Some of these compounds require a relatively large substituent to be modeled in, leading to uncertainty in the protein and ligand conformations and in the position of the compound, particularly when the fragment fits tightly in the hydrophobic binding sub-pocket and adaptation of the protein structure is necessary. The 40 ns MD equilibration carried out might not be sufficient for achieving an optimal ligand-protein configuration, which may affect the computed residence time.

Another possible reason can be deduced from the observation that sets of compounds with different buried fragments R_2_ demonstrate inhomogeneous deviations from the general linear fitting of the complete set, while sets of compounds with the same buried fragment show very similar deviations. This implies the systematic omission of a specific contribution to the observed residence time. In RAMD, conformational changes of the protein induced by the ligand's motions on the nanosecond timescale of the simulations are captured rather well, but the longer time scale motions of the protein are not fully sampled and these can be expected to modulate the ligand dissociation times. For example, if backbone changes, such as the unfolding of a helix, are needed for ligand egress, then this is likely to be captured to a lesser extent than side chain rotations in RAMD simulations. Such long-time motions may facilitate ligand dissociation, and therefore poor sampling of these motions may result in the overestimation of residence times with the τRAMD procedure.

### Elucidation of the Molecular Features Affecting Residence Time From Simulated Ligand Dissociation Trajectories

As discussed above, the relative τ value is obtained in the τRAMD procedure from the computed ligand dissociation times that are assumed to be longer for the slower dissociating compounds and shorter for the faster dissociating ones. By building a feature set of protein-ligand IFs from the ligand dissociation trajectories, we deliberately omitted information on the trajectory length (see section Methods and Materials). Instead, we assessed whether the pattern of protein-ligand contacts in the ligand dissociation trajectories contains information on the ligand dissociation mechanism and whether it can be used to deduce how ligand substituents affect residence time prolongation.

**Figure 5:**
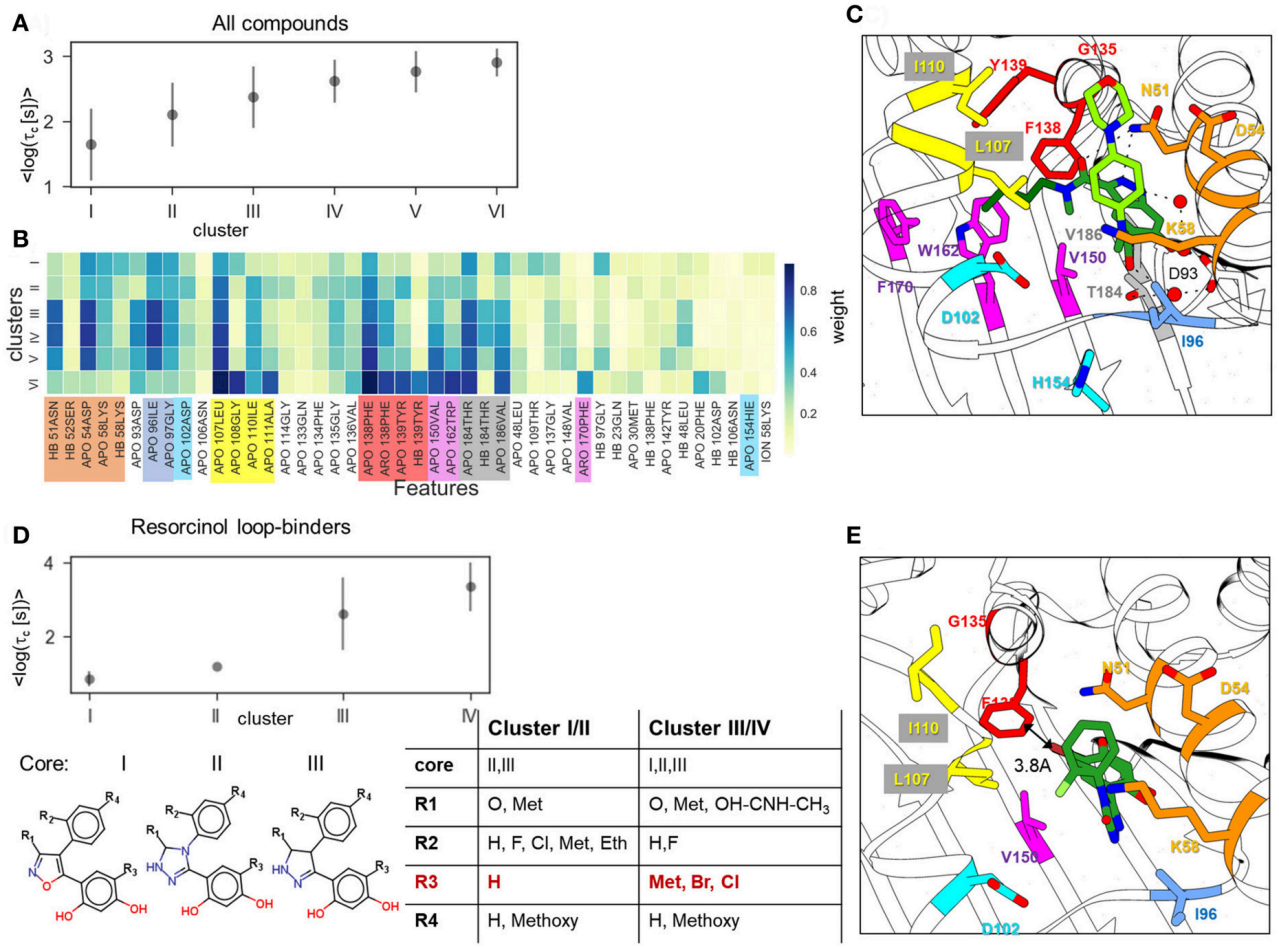
Result of clustering analysis based on the IFs of the ligand dissociation trajectories. **(A,B)** Clustering of the complete data set of 94 compounds: **(A)** mean and standard deviation of log residence times in each cluster obtained in 50 clustering runs; **(B)** weights of IFs for each cluster. HB, ION, ARO, and APO mean hydrogen bond (donor or acceptor), ionic, aromatic, and apolar interactions, respectively; **(C,E)** Position of indazole compound bound to the helix-type conformation of the binding pocket (PDB ID:5LNZ), and **(C)** of resorcinol compound bound to the loop-type conformation (PDB ID: 5J2X) **(E)**; residues that contribute to the protein-ligand contacts along the ligand dissociation trajectories are shown in stick representation and colored by protein region consistently with **(B)**. **(D)** Clustering of the resorcinol loop-binders (see compound list in Supplementary Table 2) showing mean and standard deviation of the log residence time in each cluster (above) and cluster composition (below).

To explore this, we employed the largest data-set, A, for clustering of all 94 compounds by the similarity of their IF features. We found that the optimal number of clusters was 6 (see Methods and Materials for details). Although in some clusters, the distributions of residence times are quite wide, there is a clear difference in their mean residence times, so that the clusters can be ranked by their mean τ value, τ_c_ (see Figure 5A). The average cluster properties obtained from 50 repeated clusterings mainly reflect the general structural similarity of compounds. The composition of the clusters and their order is mostly preserved in all 50 clustering rounds: the cluster with the longest average residence time comprises compounds with a tricyclic fragment, whereas the two clusters with the shortest average residence times consist mainly of loop-binders and fast unbinding compounds, such as quinazolines; in the two intermediate clusters, one contains indazoles and one contains resorcinols. From the IF weights in each cluster (Figure 5B), one can see that most of the contacts associated with large τ_c_ values arise from residues lining the hydrophobic sub-pocket formed due to α-helix3 stabilization: specifically, residues that belong to α-helix3 (L107- A111, marked in yellow in Figure 5B), those located in the hydrophobic sub-pocket at its entrance (F138, Y139, V150, W162, F170, shown in red and magenta in Figure 5B), and two residues at the bottom of the ATP binding pocket (V186 and T184, highlighted in gray). These residues are shown in Figure 5C in the same color as in Figure 5B. It is noteworthy, that the weights of several residues located at the entrance of the hydrophobic sub-pocket, specifically F138, V150, and L107, gradually increase with the residence time. This result agrees with the conclusion of our previous study that steric hinderance at the egress channel for compounds partially located in the hydrophobic sub-pocket is an important factor in increasing the transition state energy and thus prolonging the residence time (Kokh et al., 2018). The interaction with exposed residues lining the entrance to the ATP binding pocket (polar residues N51, D54) has a large contribution for the clusters III-V with intermediate residence times. However, they do not show a notable correlation with the residence time in this cluster splitting.

Overall, the splitting of the 94 compounds into just six clusters reveals several very general tendencies, showing that the interactions of the compound fragment located in the hydrophobic sub-pocket generally promote slower dissociation, while the interactions with exposed residues lining the entrance to the ATP binding pocket may affect the residence time, but without showing a systematic trend. Increasing the number of clusters leads to a general reduction of the residence time diversity in each cluster (see Supplementary Figure 5C), which suggests that the similarity of the IFs in dissociation trajectories does generally correlate with the residence time. However, to obtain a more detailed understanding of dissociation mechanisms, one has to consider clustering of specific compound sub-sets. For example, clustering of the 11 resorcinol-based loop-binders from cluster I effectively separates the faster dissociating compounds from the slower dissociating compounds (Figure 5D). Interestingly, although the cluster composition varies during repeated clustering, the main difference between the slower dissociating compounds (clusters III and IV) and the faster dissociating ones (cluster I and II) is retained: either a halogen (Cl or Br) or an aliphatic fragment (for example, a methyl group) on the resorcinol group (fragment R3 in Figure 5D) is always associated with longer residence time. All other fragments (R1, R2, and R4) appear in both groups with short and long residence times (clusters I/II and III/IV, respectively). We therefore surmise that the interaction with F138 (in particular from the Cl atom) is one of the important factors for prolongation of the residence time even though this interaction is not clearly established in the bound state (see structure shown in Figure 5E).

**Figure 6:**
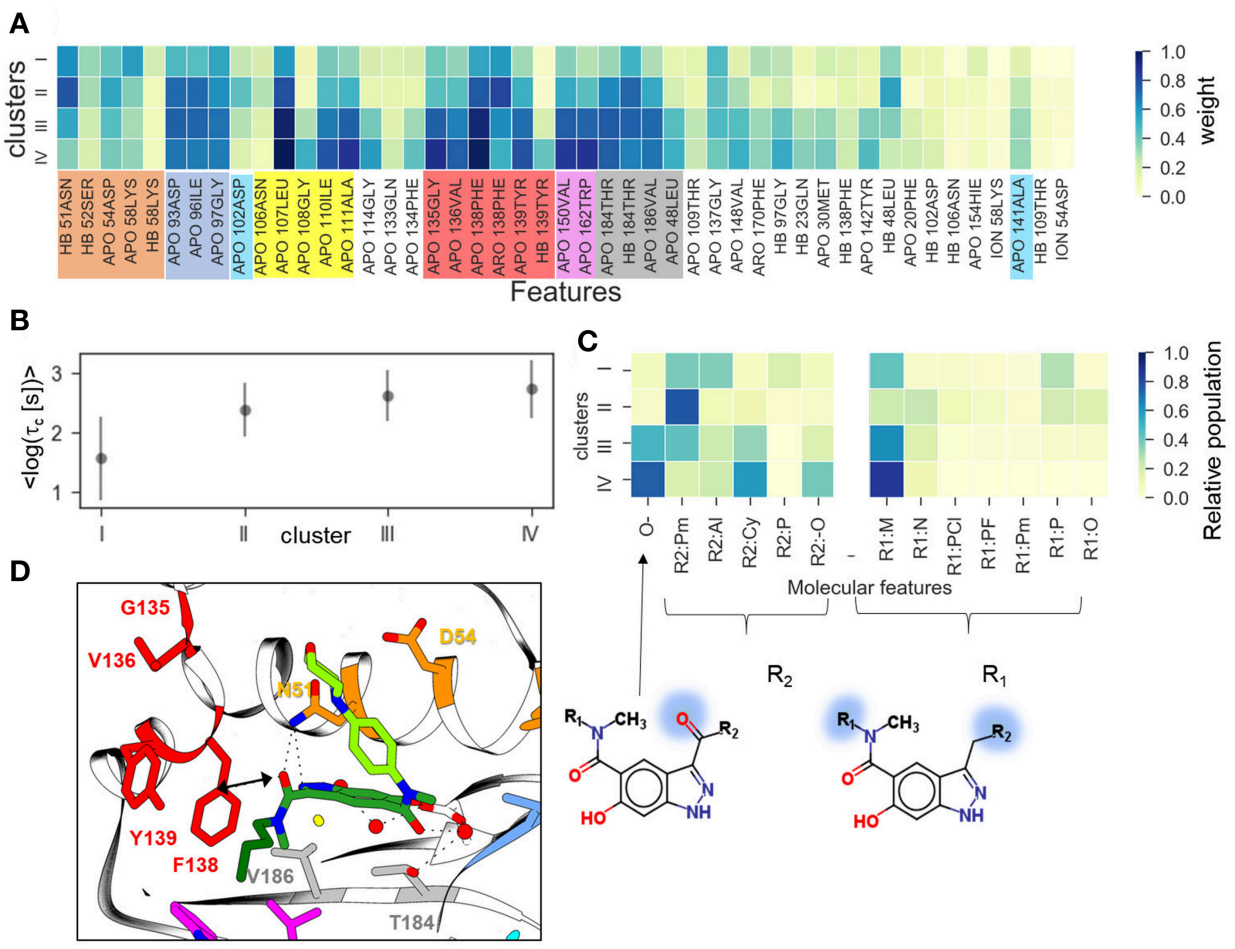
Clustering of indazole compounds: **(A)** weights of IFs for each cluster (coloring scheme and labels as in Figure 4); **(B)** mean and standard deviation of log residence times of compounds in each cluster; **(C)** population of selected molecular fragments in each cluster (see Supplementary Figure 6 for naming convention); the structures of two compounds discussed in the text are shown below (fragment substitutions are highlighted in blue); **(D)** Position of indazole compound **37** in the binding pocket, the main contact residues are shown in sticks and colored as in **(A)**.

Furthermore, we have performed clustering on the largest subset of compounds available (indazole compounds bound to the helix-type conformation). The averaged weights of different types of IFs that distinguish the four clusters are shown in Figure 6A. The mean residence time variation over the clusters (Figure 6B) shows that there is a significant gap between the fastest dissociating compounds in cluster I and the slower dissociating ones in clusters II-IV. As we observed for the complete set of compounds, the slowest dissociating clusters are characterized by a large contribution of the IF from residues lining the hydrophobic sub-pocket located at α-helix3 (L107, G108, I110, A111) or at the entrance of or inside the hydrophobic pocket (F138, V150, T139, W184). Additionally, residues G135 and V136, located between the entrance to the hydrophobic sub-pocket and the ATP binding pocket, contribute (Figure 6D). These residues may interact with the solvent-exposed part, R_1_, of the ligand, a 4-(40morpholinyl) phenyl fragment (see Figure 1D). To obtain a more detailed understanding of these protein-ligand interactions, we selected several molecular fragments that predominantly define structural variance in the indazole set (see Supplementary Figure 6) and computed the average occurrence of these fragments in each cluster (Figure 6C). It can be seen that all compounds with a carbonyl oxygen at the R_2_ fragment (located between N51 and F138 in the bound complex, see Figure 6D), belong to the long-residence time clusters III and IV. On the other hand, although N51 can form an H-bond with the carbonyl oxygen, this interaction does not have a large contribution to the slowest unbinding clusters (see Figure 6A). The results suggest that the carbonyl oxygen plays a similar role to the halogen atom in the loop-binders discussed above, and forms transient interactions with F138. Also, all compounds with alicyclic (and methoxy) groups in the hydrophobic binding pocket (indicated in Figure 6C as R2:Cy and R2:O, respectively) appear in the clusters with the longest residence times. Consistently, the hydrogen bonding (HB) interaction with the buried Y139 appears only in the slowest dissociating cluster and can be associated with a polar (carboxyl) group at the R_2_ fragment. Finally, the effect of the exposed R_1_ fragment on the residence time is less well-defined than the buried R_2_ fragment (apart from a large contribution of the 4-(40morpholinyl) phenyl fragment, R1:M, which is present in about half of the indazole compounds).

### Regression Models for the Prediction of Residence Time

**Figure 7:**
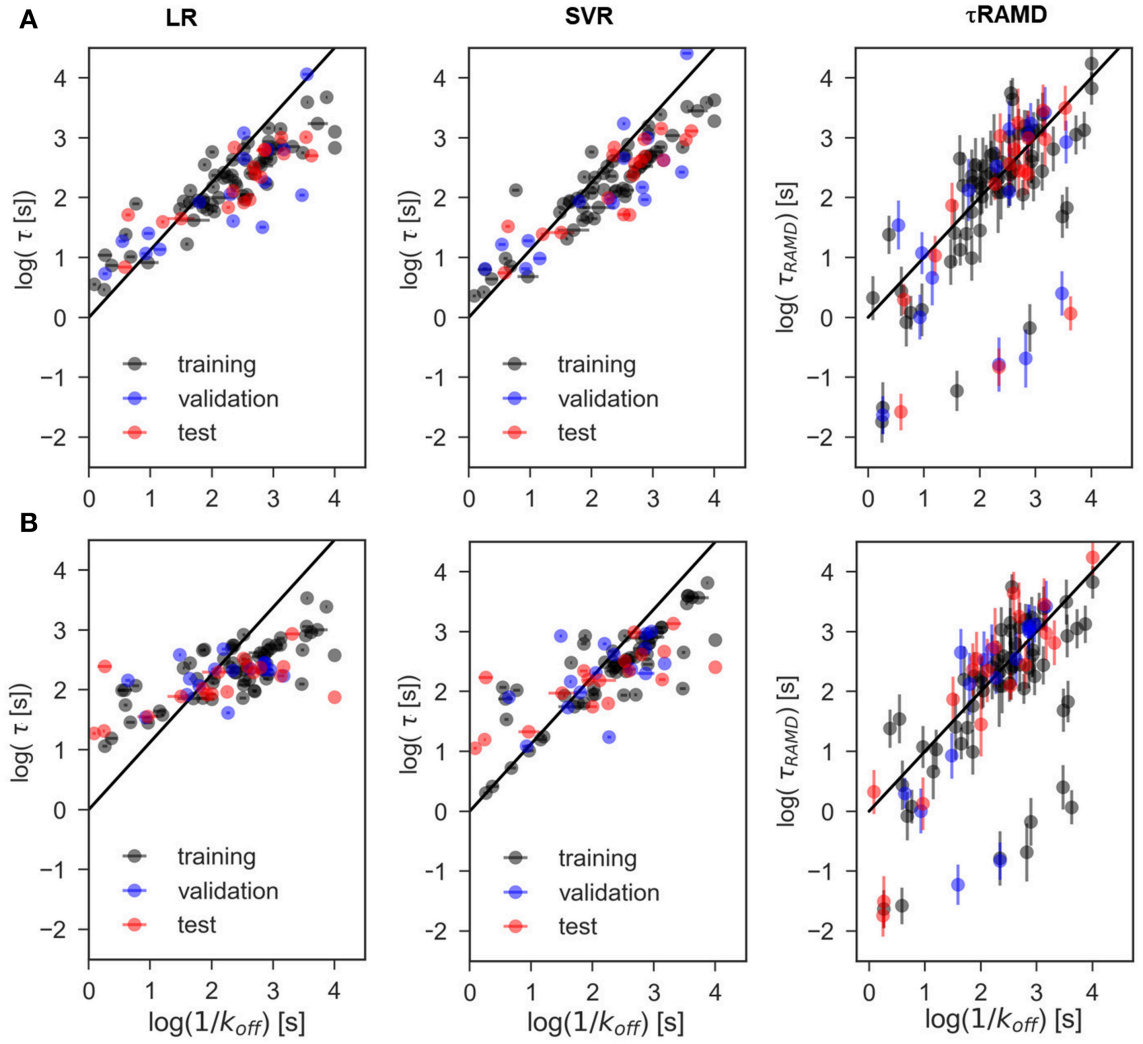
Representative examples of computed vs. experimental residence times obtained for data-sets. **(A)** A and **(B)** C using linear (LR) and non-linear (SVR) ML models as well as from the τRAMD residence time estimation procedure. Black/blue and red points belong to the training/validation and external test sets, respectively.

**Figure 8:**
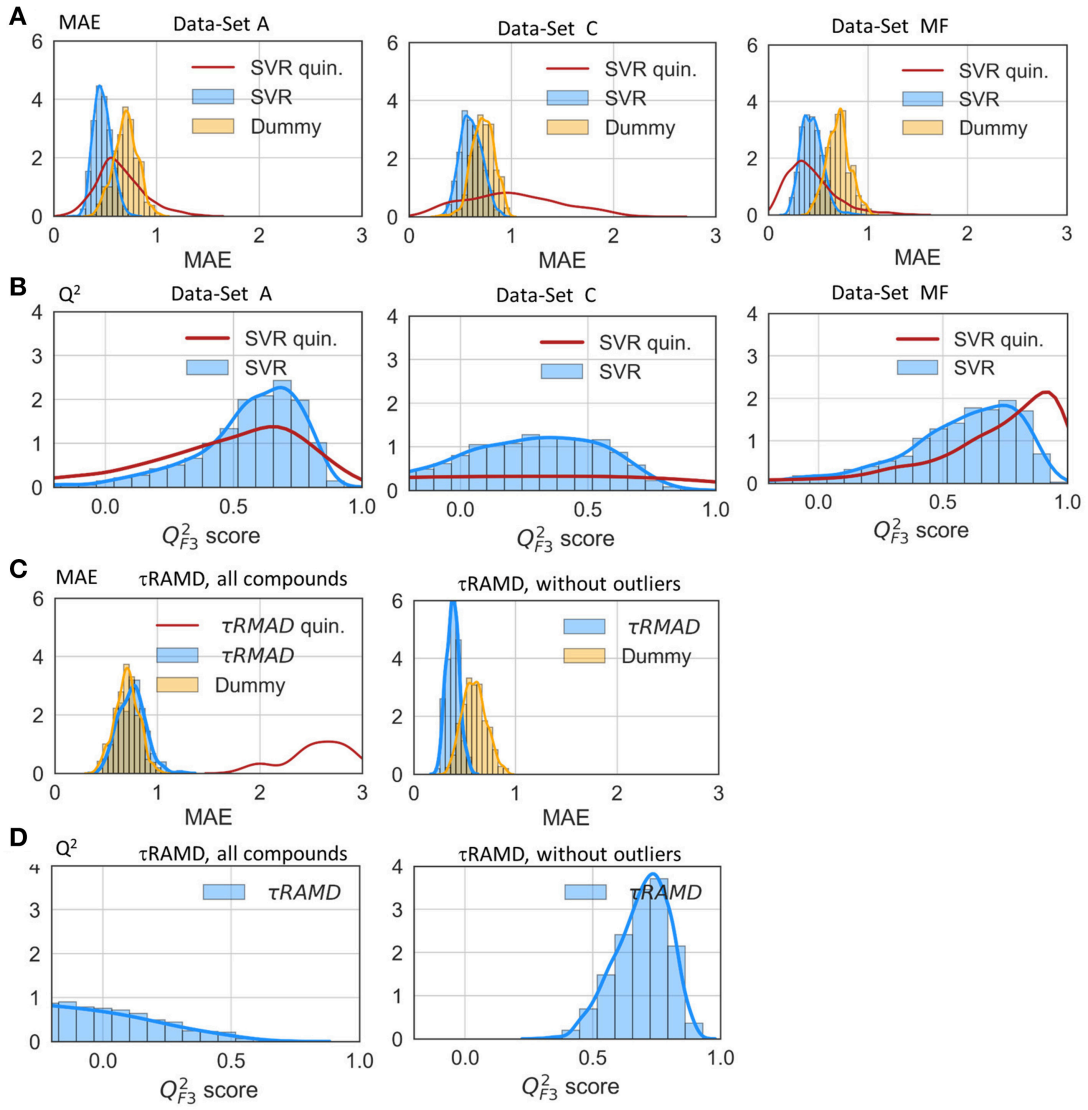
Assessment of the RM quality. Histograms of mean absolute error, MAE **(A)** and ${\text{Q}}_{\text{F}3}^{2}$ score **(B)** of the external test set obtained in 200 repeated test/training set splitting using RMs and the same values computed from τRAMD simulations **(C,D)** are shown in blue along with results for the Dummy model (orange); results for the sub-set of only quinazoline compounds (from the full data set A) are shown by red lines; in τRAMD simulations ${\text{Q}}_{\text{F}3}^{2}$ values **(D)** are negative for quinazoline compounds; in the right-hand plot of panels **(C,D)** all quinazoline compounds were removed as outliers. The data-set used are denoted in each plot: A and C data-sets, MF—data-set from molecular descriptors only.

The results of two regression models, Linear Regression with a regularization term (LR) and Support Vector Regression (SVR), to different data-sets are shown in Figures 7, 8, and the computed model quality metrics are given in Table 1. In particular, Figure 7 shows representative plots of computed against experimental residence times for the data-sets A and C. The linear and non-linear regression methods provide very similar results. Moreover, the predictions of the two methods were strongly correlated (similar under- or over-estimation of the residence times), which indicates that the data set quality, not the complexity of the RM chosen, poses the main limitation on the accuracy. Consistently, the MAE distributions for both methods obtained from 200 different test sub-sets are similar, as shown in Figure 8. The mean MAE value for the test sets are about 0.47 ± 0.08 for both RMs, while the Dummy model yields 0.71 ± 0.11 (see Table 1; the MAE histogram for the training and validation sets are shown in Supplementary Figure 7). The predictions have a ${\text{Q}}_{\text{F}3}^{2}$ = 0.57/0.56 ± 0.2 for LR and SVR RMs, respectively, which indicates that the model quality is acceptable, albeit with a relatively large standard deviation. Note, that in this model we included all compounds, even those that were considered as outliers in τRAMD simulations in Kokh et al. (2018) and each test set was required to contain at least 2 quinazoline compounds, whose τ is strongly underestimated in τRAMD simulations, as can be seen in Figure 4A. Therefore, the τ estimated directly from the τRAMD simulations has a large mean MAE of 0.76 ± 0.12 (the MAE distribution is shown in Figure 4C).

**Table 1:** Results of evaluation tests for different models: mean of MAE and ${\text{Q}}_{\text{F}3}^{2}$ score obtained from 200 rounds of simulations (the standard deviation is given in parentheses) for the external test sets.

	**RM**	**A**	**B**	**C**	A*	**MF**	**Ind**
MAE	LR	0.47(0.08)	0.51(0.09)	0.60(0.11)	0.43(0.08)	0.51(0.10)	0.39(0.10)
	SVR	0.48(0.09)	0.53(0.10)	0.60(0.11)	0.43(0.08)	0.45(0.11)	0.39(0.11)
	τRAMD	0.76(0.12)	0.39(0.06)	–	0.38(0.08)		
	Dummy	0.71(0.11)	0.61(0.11)	0.71(0.11)	0.55(0.14)		
${\text{Q}}_{\text{F}3}^{2}$	LR	0.57(0.21)	0.44(0.30)	0.29(0.30)	0.54(0.23)	0.36(0.52)	0.41(0.52)
	SVR	0.56(0.22)	0.44(0.30)	0.28(0.30)	0.51(0.25)	0.52(0.30)	0.38(0.58)
	τRAMD	−0.41(0.47)	0.69(0.10)	–	0.57(0.23)		

Calculations were done for data-sets A, B, and C (see main text) are based on the complete set of 94 compounds. The test sets in these three cases were required to contain some of the outliers found by applying the τRAMD procedure to estimate relative residence times, see Methods for details. A^*^–data-set of 80 compounds with outliers discarded. MF—based on molecular property features only. Ind—only IFs of indazole compounds from data-set A are included. For data-set A, the quinazoline compounds (8 compounds) have a mean MAE = 0.60 ± 0.2/0.61 ± 0.2 and ${\text{Q}}_{\text{F}3}^{2}$ = 0.44 ± 0.4/0.41 ± 0.4 for LR and SVR models, respectively; for the data set MF quinazoline compounds have a mean MAE = 0.59 ± 0.21/0.43 ± 0.25 and ${\text{Q}}_{\text{F}3}^{2}$ = 0.45 ± 0.39/0.65 ± 0.42 for LR and SVR models, respectively; for the Dummy model ${\text{Q}}_{\text{F}3}^{2}$ = 0.

**Figure 9:**
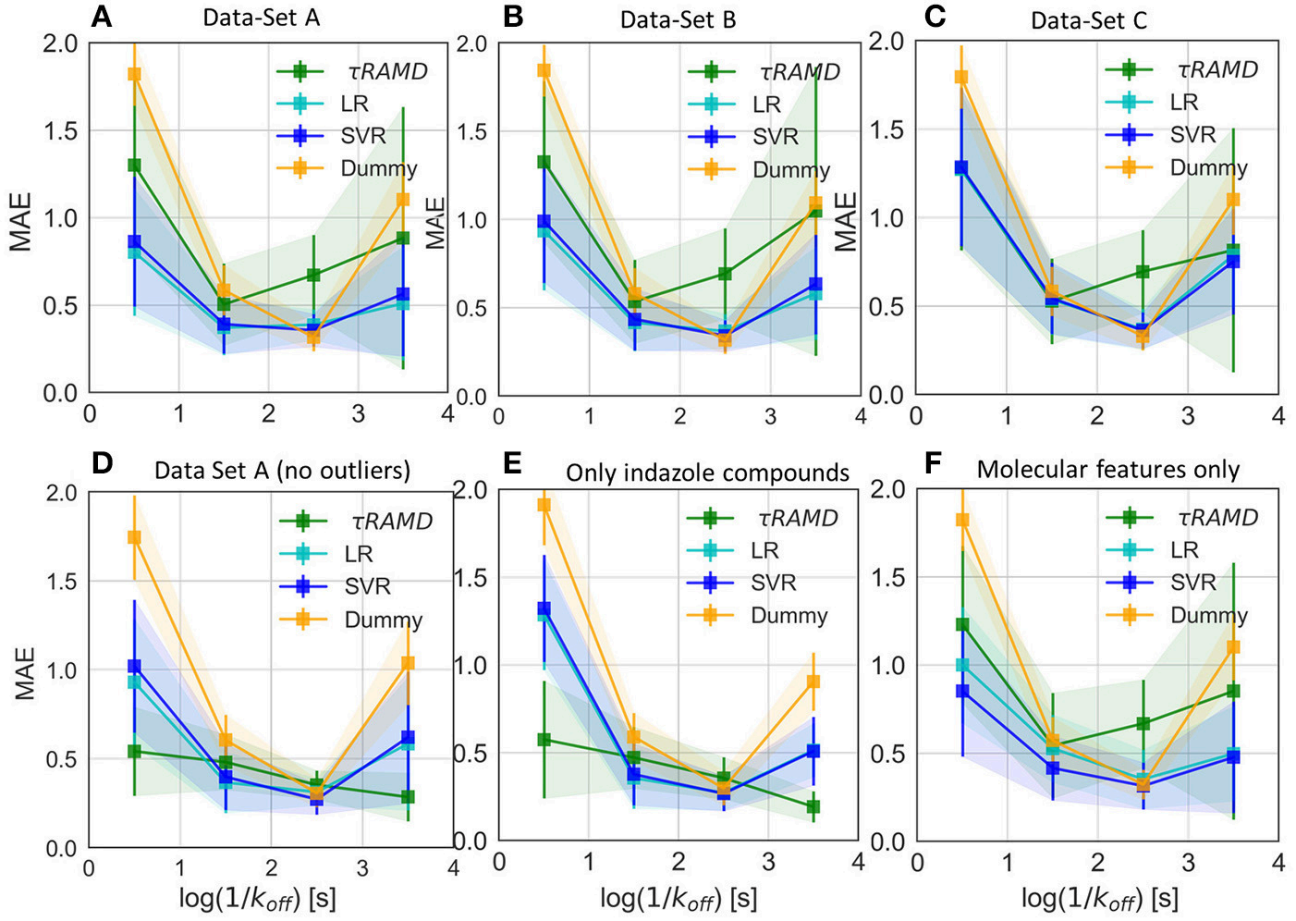
Average value of MAE for the sub-set of compounds with experimental residence times in the ranges of (<1s), (1s-2s), (2s-3s), and (>3s) as obtained in 100 simulations for different test sets and compared with the Dummy-model (null-hypothesis) and τRAMD for the same set of compounds. **(A–C)** For the complete set of compounds in models **(A–C)**, respectively; **(D)** For the data-set model A* (model A without outliers); **(E)** Only a sub-set of indazole compounds from the data-set A was used; **(F)** Only molecular features were used.

To gain deeper insight into the determinants of the quality of the RMs, we split the τ interval into four regions and plotted the mean of the MAE distributions for each region (Figure 9). Both RMs have almost identical results and they clearly outperform τRAMD for all four intervals used if all the compounds are considered (Figure 9A). However, the τRAMD method yields better prediction accuracy than the RMs for the shortest and longest residence time intervals if the 14 outliers (highlighted in Figure 4A) are not included in the compound set (Figure 9D, data-set A without outliers), with a mean of MAE = 0.39 ± 0.06 and ${\text{Q}}_{\text{F}3}^{2}$ = 0.69 ± 0.10, see Table 1. On the other hand, the quality of the RMs is only slightly changed on removal of the outliers, see Table 1. This is likely due to the much larger number of ligands with intermediate τ values than those with short or long τ, as can be seen from the histogram in Figure 4A, which ensures better training of RMs in the middle of the interval but difficulties in the prediction of more extreme values.

To further assess the ability of the RMs to correctly predict the residence times of the compounds that appear as outliers in τRAMD simulations, we computed the MAE distribution for a test subset consisting of quinazoline compounds only, which yielded a mean value of MAE = 0.60 ± 0.2 (MAE distribution from the model dataset A is shown by a red line in Figure 8) and a mean ${\text{Q}}_{\text{F}3}^{2}$ = 0.44 ± 0.4. This result is worse than for the whole set of compounds, probably because of the small number of quinazoline compounds in the training set: 6, and in the external test set, 2. Nonetheless, the estimation of τ from RMs is much better for these compounds than that obtained from τRAMD simulations of the residence time based on the trajectory length, which results in underestimation of τ by several orders of magnitude. This is an important result suggesting that the residence time can be reasonably well-predicted by RMs trained on diverse compounds whereas τRAMD simulations cannot always be used to rank τ computed for compounds with different scaffolds. In Kokh et al. (2018), it was hypothesized that the main reason for the underestimation of the residence time of the quinazoline compounds in τRAMD simulations was the deficiency of the bound state representation in MD simulations. Following this hypothesis, one may assume that the robustness of ML models for such compounds is a consequence of the data preprocessing, where the major part of the trajectory in which the bound-state is sampled is discarded (i.e., the main bound-state IFs are still considered but the exact length of the bound-state trajectory is not retained).

To explore the importance of the bound state IFs for RMs, we applied the same protocol using trajectories starting from snapshots where 20% and 60% of the bound-state contacts were lost (model data-sets B and C, respectively), which corresponds to loss of 2–3 and 5–16 contacts, depending on the compound size. Data-set B yielded only slightly worse prediction accuracy than data-set A, whereas the predictive ability for data-set C was notably worse and closer to the null hypothesis (see Figures 7–9 and Table 1), especially for compounds with short residence times, Figure 9C. The ${\text{Q}}_{\text{F}3}^{2}$ score of the RMs drops from 0.57 to 0.44 and then to 0.29 for the data sets A, B, and C, respectively, with SD values increasing, indicating a strong dependence of the model performance on the test subset selected.

**Figure 10:**
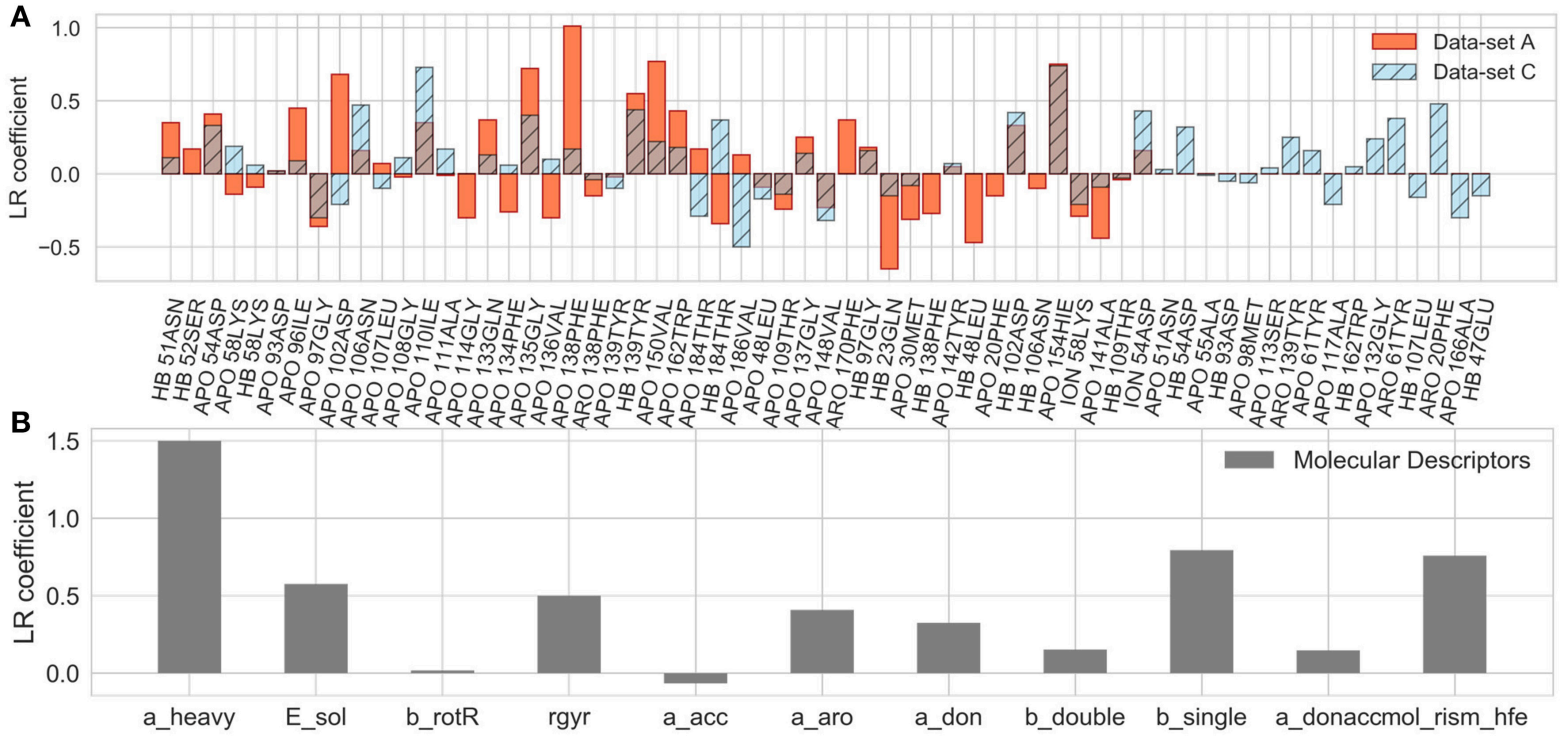
Coefficients of the LR model in the test set averaged over 200 different splitting of the training and external test sets for the A and C data-sets **(A)** and for the LR built on molecular descriptors only **(B)**, as denoted in each plot.

The coefficients of the IFs in the LR model on the data-set A and C are compared in Figure 10A. The features that have major contributions are quite similar for the data-sets A and B (data for the set B are not shown). The largest contribution comes from several residues lining the binding pocket and located at the entrance of the hydrophobic sub-pocket (F138, V150, G135), which is generally consistent with the clustering analysis given above. Additionally, several more distant residues, such as D102 and H154, appear to be important for the LR model. It is noteworthy that in both the clustering analysis and LR, the interaction with F138 plays a major role and correlates with longer residence times. For the data-set C, however, the hydrophobic sub-pocket residues do not contribute essentially. Instead, the role of polar residues around the pocket entrance (D54, N106, K58) and more distant residues, such as I110 and T61, or even F20 (located at the exit of the hydrophobic sub-pocket) increases. These results suggest that: (i) the presence of the bound state IFs in the feature set is crucial for the quality of RMs for prediction of residence times, although the RMs do not seem to be very sensitive to the exact duration of the bound state, (ii) dissociation pathways may be very diverse, which makes it difficult to build a consistent model from transition state information only.

Notably, the residues that make the main contributions to the LR and to the clustering models in the present study are quite similar to those reported for COMBINE analysis of HSP90 inhibitors (Ganotra and Wade, 2018). They include residues of the part of the α-helix3 fragment that lines the ATP binding pocket (L107-A111), as well as some polar residues surrounding the ATP binding site (N51, D54, D93, G97, D102), and several residues inside the hydrophobic sub-pocket (Y139 and T184). This agreement supports the main trend in the dissociation kinetics of the HSP90 inhibitors studied, namely that large compounds that bind in the hydrophobic sub-pocket formed by αhelix3 are generally slower dissociators. The importance of the interaction of the ligand with F138 was not highlighted by the COMBINE analysis, likely because this residue does not always directly interact with the ligand in the bound state. On the other hand, some polar residues, such as K58, N51, and D54, seem to have less importance when the complete dissociation trajectory is considered. For example, although a H-bond between some ligands and K58 is observed in the crystal structures, it is quite unstable in MD simulations and its contribution is negligible to both the LR and the clustering models.

RMs built for the congeneric series of 45 indazole compounds (data-set Ind) demonstrate similar performance for the mid- and long-range residence times to those for the complete data set (Table 1, Figure 9E and Supplementary Figure 8). For the region with k_off_ > 0.01 s^−1^, however, the model quality is poor because only 3 indazole compounds belong to this region.

Finally, we considered whether the model could be improved by the inclusion of parameters describing the molecular features of the ligands or even by training the model solely on ligand parameters. Thus, we added several molecular descriptors, such as solvation energy, number of heavy atoms, single, double and aromatic bonds, hydrogen donors and acceptors, and radius of gyration (see Supplementary Table 2) to the set of IF features. Although the RMs were not notably improved (data not shown), the number of heavy atoms appeared as a major term in the LR model. We therefore went further and trained RMs on molecular descriptors alone. Surprisingly, the SVR model based on just molecular descriptors demonstrated a good performance (${\text{Q}}_{\text{F}3}^{2}$ = 0.52 ± 0.30), comparable to that for data-set A, albeit with a larger SD, and better than the LR model (${\text{Q}}_{\text{F}3}^{2}$ = 0.36 ± 0.52) on the same dataset (see also MAE and ${\text{Q}}_{\text{F}3}^{2}$ histograms in Figure 9F). The latter is mostly driven by the number of the heavy atoms in the molecule (Figure 10B), which is an expected result since there is a clear correlation between the residence time and the number of heavy atoms (R^2^ = 0.74, Supplementary Figure 9A). The number of single bonds and solvation energy are the next most important factors, where the dependence on the solvation energy is mostly driven by the compounds with different buried fragments, in particular, indazole compounds (Supplementary Figure 9C) while variation of the exposed fragment does not have much effect (the correlation of solvation energy with log(1/k_off_) for different sub-sets is shown in Supplementary Figures 9B–F).

## Conclusions

In the present study, we propose a protocol for estimating drug-target residence times and for exploring which protein-ligand interactions affect the residence time. We performed a machine learning analysis of ligand dissociation trajectories obtained from τRAMD simulations. For the evaluation of the method, we analyzed the ligand dissociation trajectories of 94 inhibitors of HSP90 [previously published for 69 compounds (Kokh et al., 2018) and simulated for an additional 25 compounds from Schuetz et al. (2018b)]. We excluded from the analysis the first part of each simulated trajectory where the majority of protein-ligand interactions were retained as in the starting complex structure. We considered three different thresholds for defining the minimum number of protein-ligand contacts that must be lost to assign a snapshot to the transition part of the trajectory: (i) 2 contacts, (ii) 20%, and (iii) 60% of all bound-state contacts (data-sets A, B, and C, respectively). A collection of protein-ligand interaction fingerprints, IFs, extracted from the transition part of each dissociation trajectory as defined above, was employed to build a set of features for machine learning analysis.

We first explored the possibility to obtain insights into key protein-ligand contacts and to reveal ligand fragments that influence the ligand residence time using a clustering algorithm and the data-set A. Then, we built regression models, RMs, for the prediction of ligand dissociation rates using experimental data. We tested different data models, as well as a data sub-set containing indazole compounds only, and a set of molecular descriptors. We systematically compared the predictive performance of the RMs with the null-hypothesis, as well as with the results of the τRAMD method, where relative residence times were estimated based on the lengths of the dissociation trajectories for each compound. We found that RMs have good predictive ability for residence times, even for compounds where the τRAMD method fails because of deficiencies in the modeling of the ligand-protein bound state due to force field or sampling issues.

Comparison of the three data-sets, with different definitions of the transition part of the trajectory, shows that the residence time strongly depends on the interaction of the ligand with residues of the binding cavity, when most of the bound state protein-ligand contacts are still preserved. This is in accord with the recent calculations of relative residence times for HIV-1 protease inhibitors (Huang et al., 2019) and HIV-1 protease and HSP90 inhibitors (Ganotra and Wade, 2018), which demonstrated that protein-ligand contacts in the complex could be used to deduce ligand residence times. From the linear regression model, as well as from clustering analysis, we found out that the interaction of the ligand with F138 is very important. Although F138 is not always directly contacting the ligands in their bound states, it forms transient interactions with aromatic groups as well as with polar groups of the binding core (either halogen or carbonyl oxygen) present in most of the compounds, and thereby promotes prolongation of the ligand residence time.

As expected, the quality of the ML models strongly depends on the range and the homogeneity of the distribution of kinetic rate constants for the compounds studied, and the size of the set of compounds with similar scaffolds but different substitutions. In particular, the quality of the present models is strongly affected by the fact that about 50% of the compounds have intermediate residence times, while there are much fewer compounds with short or long values of τ.

Finally, we demonstrated that the LR model based only on the molecular features of the compounds reproduced the general trend in τ reasonably well. It showed an increase of τ with molecular size, but was less reliable for the prediction of the dissociation rates of compounds with short τ values, for which the determinants of the dissociation kinetics are more complex. On the other hand, the SVR model trained on the molecular features shows surprisingly good performance (similar to that obtained when the model was trained on the complete set of IFs), albeit with a larger variation in the performance for different sub-sets of compounds.

Overall, this study demonstrates that the proposed machine learning procedures can effectively extend the value of the τRAMD procedure by making corrections for outliers, improving the predictive ability for ligand residence time, and giving information on key determinants of the ligand dissociation mechanism and the ligand functional groups that are critical for residence time prolongation.

## Data Availability

The IF data set and Python Jupyter Notebook scripts and a data set are accessible at https://zenodo.org/record/2652166#.XMMg6YWxU5k (doi: 10.5281/zenodo.2652166), the raw data are available from the authors upon request, without undue reservation, to any qualified researcher. Kinetic data can be found in Kokh et al. (2018), Schuetz et al. (2018b), and Amaral et al. (2017) along with the Protein Databank identifiers of crystal structures of protein-ligand complexes. The 2D structures of the compounds used in the study are given in SMILES format in the Microsoft Excel Supplementary Table 4 as a separate file.

## Author Contributions

DK and RW conceived and designed the study. DK and BK carried out the MD simulations. DK, TK, and BK performed the machine learning analysis. DK wrote the first draft of the manuscript. All authors contributed to manuscript revision, and read and approved the submitted version.

### Conflict of Interest Statement

The authors declare that the research was conducted in the absence of any commercial or financial relationships that could be construed as a potential conflict of interest.
