# Supplementary material for: Machine Learning Analysis of τRAMD Trajectories to Decipher Molecular Determinants of Drug-Target Residence Times
Source: Front Mol Biosci. 2019 May 24;6:36. doi: 10.3389/fmolb.2019.00036 (PMC6543870; doi:10.3389/fmolb.2019.00036)
Supplement: Supplementary file 1 [file Data_Sheet_1.pdf]

Supplementary Material

1 Supplementary Data

1.1 Optimization of hyperparameters in regression models

For hyperparameter optimization, we used an exhaustive grid-search and 10-fold cross-validation using random permutation splitting with a validation set of the size of 20% of the data. The hyperparameters were optimized for each training/test split.

Linear regression model with L^2 regularization (LR): For this model, the loss function is the least squares function and the regularization term is defined as the L^2-norm. The only fitting parameter is a regularization coefficient (α). In the optimization procedure, it was varied in the interval [0.01 – 100] with 20 points on a linear scale.

Support Vector Machine Regression with RBF kernel (SVR): The SVR model optimizes a linear function in the space of the kernel given by $\exp(-\gamma\|x-x'\|^2)$ and uses an epsilon-insensitive loss function, i.e. the *L1* linear loss function with a minimization threshold parameter ε. The values of γ and ε were optimized, as well as coefficient C of the regularization term. We used the following intervals for fitting: C= [0.1-300]; γ = [0.003-10]; ε = [0.1-0.6] (with 20, 15, and 10 points on a logarithmic scale and for C, γ , and ε , respectively).

The typical variation of the cross-validation score for each of the hyperparameters is shown in Supplementary Figure 2 A, B. The distributions of the hyperparameters obtained in 200 rounds of training/test set splits are shown in Supplementary Figure 3.

1.1.1 Assessment of Regression Models

Regression Model: We optimize log(1/k) values, where k is the measured rate constant (k_{off}, k_D, or k_{on}).

Mean Absolute Error (MAE): A measure of how much the predicted values deviate from the experimental ones (for this, we use a logarithmic (log10) scale)

$$MAE = 1/n\sum_{i=1}^{n-1}|y_i - \hat{y}_i|$$

where y_i is the experimental value and $\hat{y}_i$ is the computed values, where i =1…n is the set of compounds considered. For example, y_i is an element of the set of experimental $\log(1/k_{off})$ values)

Mean Prediction Uncertainty (MPU): A measure of the relation between experimental and computed residence times:

$$MPU = 1/n\sum_{i=1}^{n}\max\{10^{y_i-\hat{x}_i}, 10^{-y_i+\hat{x}_i}\}$$

$$\hat{x}_i = \hat{y}_i/a$$

where a is a slope of the linear fitting of the computed τRAMD relative residence times to the experimental values on the logarithmic (log10) scale. In the case of LR models $\hat{x}_i = \hat{y}_i$.

$\boldsymbol{Q^2_{F3}}$ **score:** A metric for the evaluation of the predictive ability of a model that has been shown to have superior mathematical properties over other metrics (Todeschini et al. 2016):

$$Q^2_{F3} = 1 - \sqrt{\frac{\sum_{i=1}^{n-1}(y_i - \hat{y}_i)^2 / n}{\sum_{i=1}^{n_{tr}-1}(y_i - \bar{y}_{tr})^2 / n_{tr}}}$$

where

$$\bar{y}_{tr} = 1/n_{tr} \sum_{i=1}^{n_{tr}-1} y_i$$

is the experimental value in the training set consisting of n_{tr} elements.

1.1.2 Clustering

Clustering using a Gaussian Mixture Model (GMM), based on the assumption that the data points have a Gaussian distribution, was performed by optimizing the mean and the standard deviation. A GMM can assign a data point to more than one cluster and provides a probability of each data point belonging to each of the clusters. The data distribution for K Gaussian distributions is defined as:

$$p(x) = \sum_{k=1}^{K} w_k G(x|\mu_k, \Sigma_k)$$

where w_k is the weight (mixing coefficient) of the data point x in the cluster k and μ_k and Σ_k are the mean and covariance, respectively, of the cluster k with a probability density function of a multivariate Gaussian distribution, G. The Expectation–Maximization (EM) algorithm is used for the optimization of the mean and variance of each cluster.

Minimization of the Akaike information criterion (AIC) was used to find the optimal model, where AIC is defined as:

$$AIC = 2k - 2n < L >$$

where k is the number of clusters, n is the size of the dataset, and $<L>$ is the log-probabilities per each component for each cluster averaged over all clusters.

2 Supplementary Figures and Tables

2.1 Supplementary Tables

Supplementary Table 1. 2D images of compounds used in the study

1 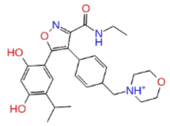	2 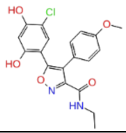	3 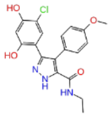	4 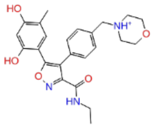	5 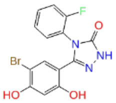
6 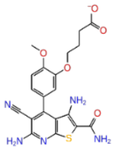	7 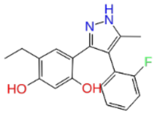	8 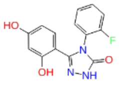	9 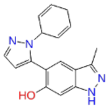	10 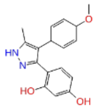
11 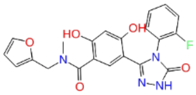	12 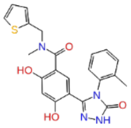	13 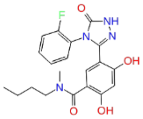	14 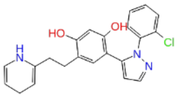	15 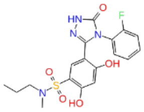
16 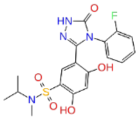	17 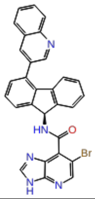	18 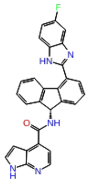	19 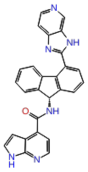	20 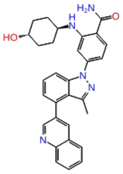
21 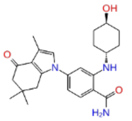	22 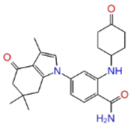	23 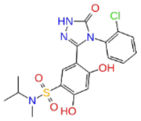	24 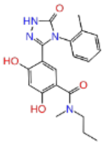	25 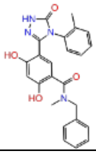
26 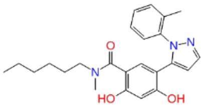	27 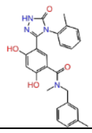	28 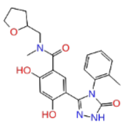	29 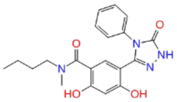	30 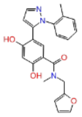
31 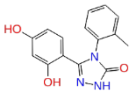	32 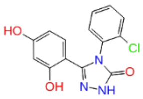	33 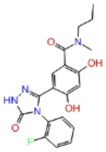	34 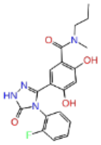	35 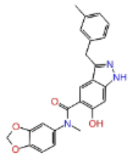

36 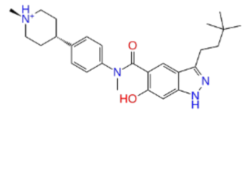	37 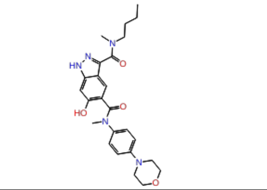	38 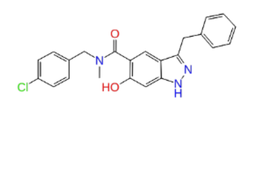	39 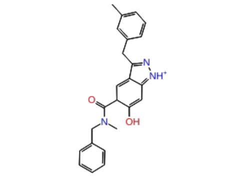	40 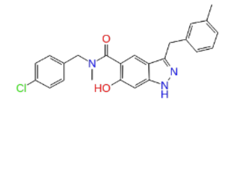
41 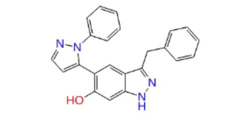	42 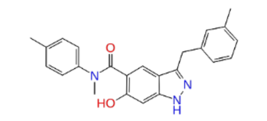	43 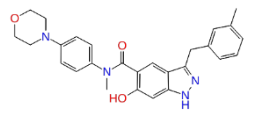	44 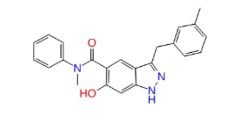	45 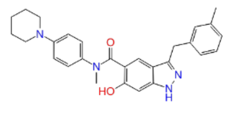
46 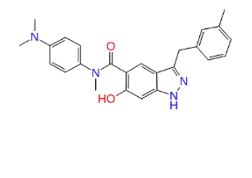	47 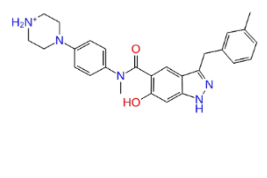	48 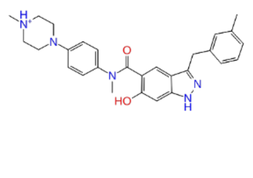	49 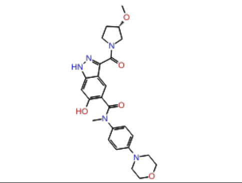	50 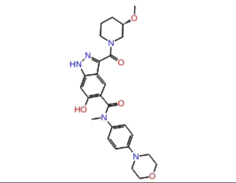
51 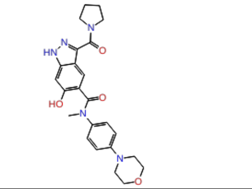	52 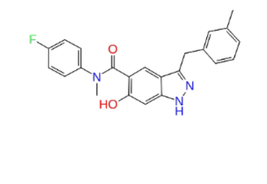	53 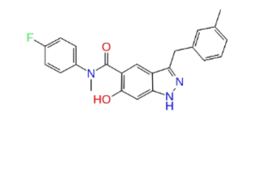	54 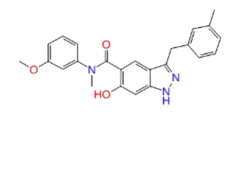	55 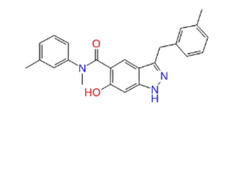
56 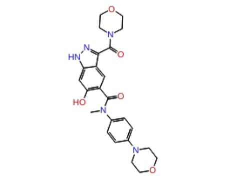	57 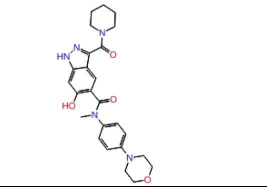	58 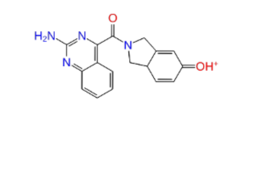	59 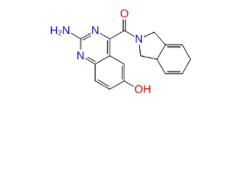	60 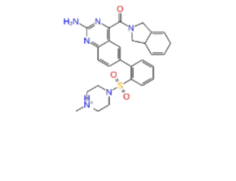
61 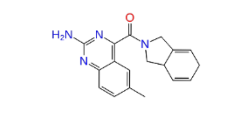	62 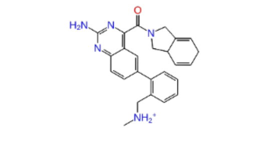	63 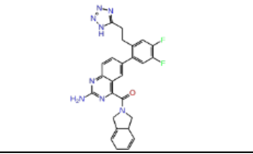	64 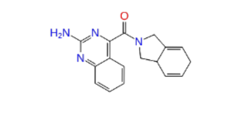	65 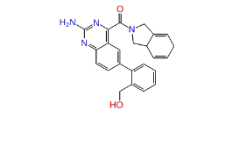
66 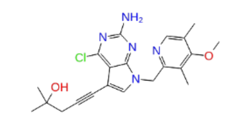	67 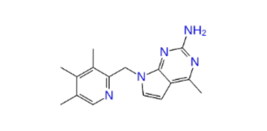	68 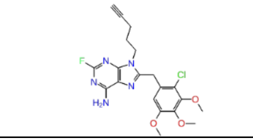	69 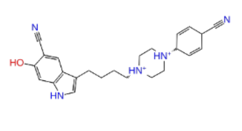	70 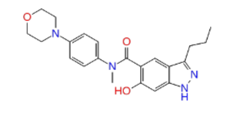
71 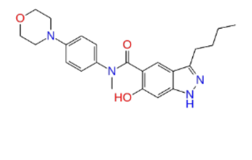	72 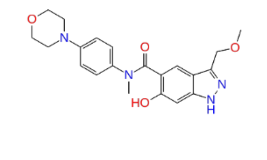	73 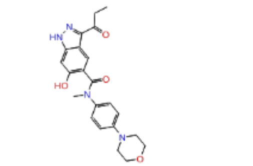	74 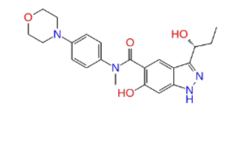	75 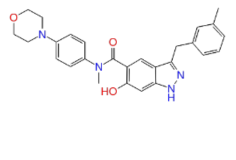

76 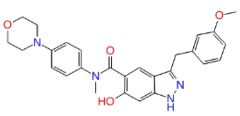	77 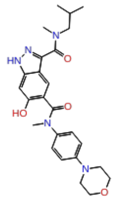	78 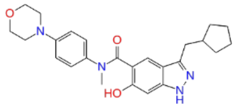	79 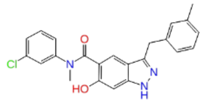	80 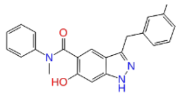
81 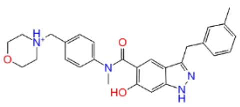	82 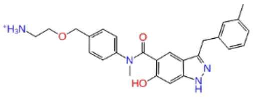	83 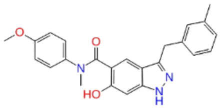	84 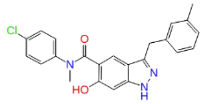	85 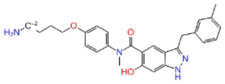
86 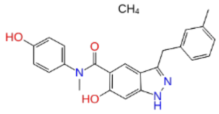	87 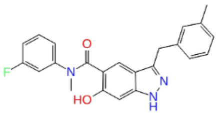	88 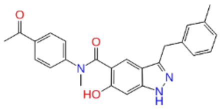	89 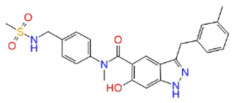	90 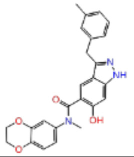
91 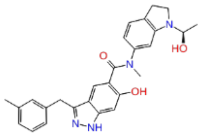	92 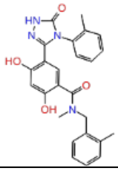	93 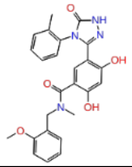	94 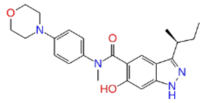	

Supplementary Table 2.

Molecular properties and kinetic data of compounds used in the study. Molecular properties were computed using the MOE software (MOE 2017). The outlier compounds as defined in (Kokh et al. 2018) are highlighted in yellow.

Compound: Identifier used in this work;

In literature: Identifier used in previous publications. The structural and kinetic data of the first 69 compounds are from (Kokh et al. 2018) and the data for the remaining 25 compounds are from (Schuetz et al. 2018);

Type: Resorcinol-, indazole -, and quinazoline -type compounds are denoted by R, I, and Q, respectively (highlighted); a, h, b, i, z, t, p – adenine, 6-hydroxyindole, benzamide,7-imidazopyridine, 7-azaindole, aminothienopyridine, aminopyrrolopyrimidine; Loop-type compounds are colored in cyan.

k_{off}, k_{off}_SD: Experimental dissociation rate constant and its standard deviation;

ramd and ramd_SD: Computed τRAMD residence time and its standard deviation;

MPU : Mean prediction uncertainty of the τRAMD simulation for a particular compound (folds of τ), see details in the Supplementary Data and in Ref. (Kokh et al. 2018);

replicas: Number of equilibration simulations used for starting for ligand dissociation simulations;

a_heavy: Number of heavy atoms in ligand;

E_sol: Ligand solvation energy;

rgyr: Ligand radius of gyration;

a_acc, a_don, a_donoracc : Number of H-bond acceptors, donors, and their sum in the ligand;

b_double, b_single, b_rotR, a_aro : Number of double, single, rotatable, and aromatic bonds, respectively, in the ligand;

mol_rism_hfe : 3D-RISM solvation energy.

Compound	in literature	Type	koff	koff_SD	ramd	ramd_SD	MPU	replicas	a_heavy	E_sol	b_rotR	rgyr [Å]	a_acc	a_aro	a_don	b_double	b_single	a_donacc	mol_rism_hfe [kcal/mol]
1	1	R	1.0E-04	0.E+00	12.9	3.4	2.5	4	34	-84	0.22	4.75	5	11	3	3	57	8	-65
2	2	R	2.0E-03	1.E-04	2.6	1.0	1	6	27	-23	0.21	4.01	5	17	3	1	28	8	-26
3	3	R	1.0E-02	9.E-04	2.2	0.6	2.1	4	27	-34	0.21	4.00	6	17	5	1	29	11	-39
4	4	R	1.0E-04	0.E+00	8.9	2.1	1	4	32	-88	0.20	4.53	5	17	3	1	45	8	-73
5	5	R	1.4E-02	2.E-03	0.7	0.2	3.8	4	22	-22	0.08	3.30	4	12	3	2	19	7	-16

6	6	t	1.1E-01	2.E-02	0.8	0.2	3.5	4	31	-154	0.27	4.37	5	15	3	2	32	8	-117
7	7	R	6.3E-02	3.E-03	0.7	0.2	1.1	5	23	-20	0.12	3.40	4	17	4	0	25	8	-16
8	8	R	2.1E-01	3.E-02	0.3	0.1	5.1	4	21	-24	0.09	3.13	4	12	3	2	19	7	-20
9	9	I	8.3E-01	5.E-02	0.4	0.1	3.7	5	22	-24	0.08	3.30	4	14	3	2	24	7	-24
10	10	R	2.5E-01	2.E-02	0.4	0.2	1.4	5	22	-34	0.13	3.56	5	11	4	2	29	9	-37
11	11	R	3.3E-04	2.E-05	1.3	0.4	33.9	6	31	-36	0.18	3.91	5	17	3	3	31	8	-21
12	12	R	1.2E-03	3.E-04	3.0	1.0	1.3	5	31	-41	0.18	3.93	5	17	3	3	34	8	-20
13	13	R	1.7E-03	5.E-04	1.8	0.4	3	5	29	-30	0.23	3.83	5	12	3	3	37	8	-21
14	14	R	1.7E-01	2.E-02	0.3	0.1	2.2	5	28	-26	0.16	3.99	3	17	3	2	32	6	-22
15	15	R	6.4E-03	4.E-04	3.3	1.2	2.8	6	29	-32	0.19	3.70	6	12	3	2	36	9	-21
16	16	R	1.4E-02	2.E-03	1.4	0.4	1.3	5	29	-30	0.16	3.58	6	12	3	2	36	9	-21
17	17	R	2.8E-04	5.E-06	1.5	0.5	29.6	3	36	-40	0.10	5.28	5	31	4	1	28	9	-34
18	18	z	1.9E-04	7.E-05	4.5	1.3	2.8	4	35	-34	0.10	5.30	5	30	5	1	26	10	-31
19	19	z	2.9E-04	5.E-05	4.0	1.3	2.6	4	34	-44	0.10	5.12	6	30	5	1	25	11	-37
20	20	b	1.4E-04	4.E-06	4.8	1.3	2.8	5	37	-27	0.12	5.39	4	25	3	1	43	7	-31
21	21	b	4.9E-04	1.E-04	3.6	1.2	1.9	6	30	-21	0.12	4.41	3	11	3	2	51	6	-24
22	22	b	7.7E-04	5.E-05	2.6	0.7	2.8	5	30	-19	0.12	4.37	3	11	2	3	48	5	-26
23	23	R	9.6E-03	5.E-04	2.0	0.8	1.7	5	29	-41	0.16	3.60	6	12	3	2	36	9	-19
24	24	R	4.7E-03	8.E-04	1.9	1.2	1.1	7	28	-28	0.20	3.66	5	12	3	3	37	8	-20
25	25	R	1.1E-03	5.E-04	2.2	0.6	2.6	4	32	-34	0.17	4.00	5	18	3	3	36	8	-21
26	26	R	1.0E-02	2.E-03	1.1	0.5	2.6	6	30	-19	0.28	4.28	4	17	2	1	43	6	-16
27	27	R	1.2E-03	1.E-04	3.3	1.3	1.8	8	33	-34	0.17	4.16	5	18	3	3	39	8	-22
28	28	R	4.6E-03	9.E-04	2.3	0.5	1.6	4	31	-30	0.18	3.94	6	12	3	3	43	9	-24
29	29	R	3.0E-03	3.E-04	1.9	0.4	1.5	4	28	-27	0.23	3.86	5	12	3	3	37	8	-22
30	30	R	3.4E-04	4.E-05	0.4	0.1	573	5	30	-23	0.18	3.86	4	22	2	1	31	6	-19
31	31	R	1.1E-01	4.E-02	0.3	0.1	3.3	4	21	-21	0.09	3.14	4	12	3	2	22	7	-19
32	32	R	1.2E-01	2.E-02	0.3	0.1	4	5	21	-21	0.09	3.14	4	12	3	2	19	7	-19
33	33	R	7.1E-02	2.E-02	0.5	0.2	1.5	6	22	-19	0.13	3.22	4	12	3	2	25	7	-20
34	34	R	4.2E-03	5.E-04	1.9	0.8	1.3	5	28	-36	0.20	3.66	5	12	3	3	34	8	-31
35	35	I	5.4E-03	4.E-04	2.2	0.5	1.6	4	31	-32	0.14	3.98	6	21	3	1	33	9	-31
36	36	I	1.4E-03	4.E-04	2.5	0.8	1.7	4	33	-102	0.19	4.45	4	15	3	1	56	7	-70
37	37	I	2.0E-03	2.E-04	4.6	1.7	3.9	4	34	-33	0.24	4.39	6	15	3	2	50	9	-39
38	38	I	2.9E-02	1.E-03	1.0	0.5	1.3	6	29	-29	0.19	3.69	4	21	3	1	29	7	-28
39	39	I	2.2E-02	8.E-04	0.8	0.2	2.1	5	29	-75	0.19	3.81	3	12	1	5	39	4	-67
40	40	I	1.7E-02	4.E-04	1.0	0.3	1.2	5	30	-31	0.18	3.92	4	21	3	1	32	7	-29
41	41	I	2.3E-01	2.E-02	0.4	0.1	1	4	28	-30	0.13	3.83	4	26	3	0	23	7	-23
42	42	I	5.0E-03	9.E-04	2.8	0.8	2.7	4	29	-28	0.16	3.98	4	21	3	1	32	7	-19
43	43	I	6.8E-04	5.E-06	6.2	2.4	1.7	4	34	-35	0.16	4.52	5	21	3	1	43	8	-39
44	44	I	9.1E-03	1.E-03	1.8	0.4	1.7	4	28	-32	0.16	3.90	4	21	3	1	29	7	-28
45	45	I	7.4E-04	9.E-05	6.4	2.5	1.9	5	34	-29	0.16	4.52	4	21	3	1	45	7	-35
46	46	I	2.4E-03	4.E-04	2.9	1.1	1.4	4	31	-39	0.18	4.17	4	21	3	1	37	7	-35
47	47	I	1.2E-03	8.E-05	5.3	1.6	3.3	4	34	-117	0.16	4.53	4	21	3	1	45	7	-86
48	48	I	7.5E-04	1.E-04	6.1	2.0	2.9	4	35	-99	0.15	4.63	4	21	3	1	48	7	-83
49	49	I	1.4E-03	2.E-04	4.5	1.3	2.4	4	35	-31	0.18	4.46	7	15	3	2	50	10	-37
50	50	I	2.9E-04	2.E-05	6.7	2.2	1.4	4	36	-34	0.18	4.59	7	15	3	2	53	10	-44
51	51	I	2.4E-03	8.E-05	3.6	1.6	2.6	5	33	-25	0.16	4.31	6	15	3	2	46	9	-32
52	52	I	1.4E-03	4.E-04	4.3	1.7	1.4	6	35	-33	0.15	4.53	6	21	4	1	44	10	-34
53	53	I	3.2E-02	2.E-02	1.6	0.5	2	4	29	-26	0.16	3.99	4	21	3	1	29	7	-18
54	54	I	1.6E-02	4.E-03	2.0	0.9	2.1	5	30	-29	0.18	3.88	5	21	3	1	33	8	-21
55	55	I	7.7E-03	5.E-03	2.4	1.1	2.1	4	29	-27	0.16	3.91	4	21	3	1	32	7	-19
56	56	I	1.3E-02	7.E-04	2.2	0.8	2.1	6	34	-28	0.16	4.36	7	15	3	2	47	10	-44
57	57	I	9.1E-04	3.E-04	4.8	2.2	2.1	5	34	-32	0.16	4.36	6	15	3	2	49	9	-38
58	58	Q	5.5E-01	2.E-01	0.08	0.03	13.2	5	23	-104	0.08	3.84	3	10	1	4	26	4	-70
59	59	Q	5.7E-01	2.E-02	0.06	0.02	37.5	5	23	-30	0.08	3.70	4	10	2	3	28	6	-22
60	60	Q	2.4E-04	6.E-05	0.31	0.07	1737	5	38	-83	0.12	4.37	5	16	1	3	54	6	-63
61	61	Q	2.6E-01	7.E-02	0.07	0.02	91.9	6	23	-25	0.08	3.70	3	10	1	3	30	4	-18
62	62	Q	4.5E-03	4.E-04	0.15	0.04	622	4	31	-88	0.14	4.21	3	16	1	3	41	4	-61
63	63	Q	1.5E-03	2.E-04	0.16	0.07	3324	6	37	-40	0.14	4.94	7	21	4	3	39	11	-33
64	64	Q	5.5E-01	5.E-02	0.07	0.02	31.2	4	22	-26	0.08	3.62	3	10	1	3	27	4	-18

65	65	Q	4.5E-03	2.E-04	0.15	0.06	1435	4	30	-43	0.12	4.28	4	16	2	3	36	6	-25
66	66	p	1.3E-03	2.E-04	0.26	0.09	557	7	29	-31	0.19	4.51	5	15	2	0	38	7	-20
67	67	p	2.6E-02	1.E-03	0.10	0.03	270	4	21	-19	0.09	3.51	3	15	1	0	26	4	-18
68	68	a	3.3E-02	1.E-03	0.69	0.24	1.8	5	30	-31	0.28	4.09	6	15	1	0	36	7	-20
69	69	h	4.3E-01	1.E-01	1.0	0.3	19.6	5	30	-244	0.24	5.84	3	9	2	2	48	5	-200
70	1a	I	2.9E-03	5.E-04	2.5	1.1	1.6	6	29	-31	0.19	4.03	5	15	3	1	41	8	-36
71	1b	I	2.7E-03	7.E-04	2.6	0.9	1.5	6	30	-36	0.21	4.15	5	15	3	1	44	8	-36
72	1d	I	3.1E-03	4.E-04	2.0	0.6	2.8	3	29	-27	0.19	4.07	6	15	3	1	39	9	-41
73	1e	I	2.9E-01	4.E-02	1.2	0.4	8.3	4	30	-38	0.18	4.00	6	15	3	2	39	9	-38
74	1f	I	2.3E-02	7.E-04	3.2	1.1	10	7	30	-35	0.18	3.98	6	15	4	1	42	10	-33
75	1g	I	6.8E-04	5.E-06	4.2	1.7	1.5	4	34	-31	0.16	4.48	5	21	3	1	43	8	-37
76	1h	I	2.6E-03	4.E-05	7.6	2.4	12.7	4	35	-28	0.18	4.62	6	21	3	1	44	9	-38
77	1i	I	1.4E-03	1.E-04	4.7	1.5	1.9	3	34	-34	0.22	4.22	6	15	3	2	50	9	-39
78	2a	I	1.6E-03	7.E-04	5.1	1.2	2.7	4	32	-38	0.17	4.26	5	15	3	1	49	8	-36
79	3a	I	2.8E-03	4.E-04	8.3	1.6	17.9	4	29	-24	0.16	3.93	4	21	3	1	29	7	-18
80	5b	I	1.2E-02	9.E-04	2.9	1.2	3.7	3	29	-23	0.16	3.89	4	21	3	1	33	7	-23
81	5c	I	3.0E-03	4.E-05	4.3	1.5	3.2	4	35	-112	0.18	4.83	5	21	3	1	47	8	-76
82	5d	I	3.0E-03	2.E-04	4.9	2.0	4.6	4	33	-120	0.25	4.43	5	21	3	1	42	8	-63
83	5f	I	4.0E-03	3.E-04	3.0	0.9	1.5	4	30	-22	0.18	4.07	5	21	3	1	33	8	-21
84	5g	I	1.4E-02	2.E-03	2.4	0.9	3	4	29	-19	0.16	3.90	4	21	3	1	29	7	-18
85	5i	I	2.1E-03	5.E-04	5.3	2.7	3.9	5	34	-334	0.27	4.50	6	21	4	1	42	10	-455
86	5k	I	3.0E-03	7.E-04	2.9	1.1	1	3	30	-27	0.16	3.96	5	21	4	1	34	9	-28
87	5o	I	2.0E-02	1.E-02	2.1	1.0	2.9	4	29	-22	0.16	3.85	4	21	3	1	29	7	-17
88	5q	I	8.9E-03	1.E-03	2.8	1.2	2.9	7	31	-30	0.18	4.10	5	21	3	2	33	8	-25
89	5s	I	4.3E-03	5.E-05	4.4	1.5	4.8	4	34	-60	0.22	4.43	6	21	4	1	40	10	-42
90	5w	I	6.3E-03	2.E-05	2.5	1.2	1.4	5	32	-21	0.14	4.04	6	21	3	1	36	9	-23
91	5x	I	5.6E-03	7.E-05	3.4	2.0	2.9	5	34	-16	0.16	4.17	5	21	4	1	43	9	-30
92	4c	R	1.1E-03	8.E-05	4.8	1.3	1.7	4	33	-33	0.17	3.94	5	18	3	3	39	8	-27
93	4f	R	1.3E-03	1.E-04	4.6	0.9	1.6	3	34	-36	0.19	4.02	6	18	3	3	40	9	-25
94	1c	I	1.6E-03	6.E-05	2.6	0.9	2.3	4	30	-31	0.18	4.05	5	15	3	1	44	8	-34

Supplementary Table 3.

Sizes of the three feature data sets. These were defined as follows by the threshold for splitting the bound-state and transition-state parts of the RAMD dissociation trajectories. The final feature datasets used for machine learning consisted of the number or retained features (last column) x the number of compounds (second column).

Data set	No. of comp-ounds	No. of Snapshots from trajectories	Number of features		
			After summing up all snapshots/ trajectories:	After feature reduction:	After elimination of feature correlations:
**A** - at least 2 bound state contacts lost	94	2 061 340	311	68	47
**B** - at least 20% or 2-4 bound state contacts lost	94	1 126 584	311	69	48
**C** - at least 60% or 5-16 bound state contacts lost, depending on the size of the ligand	94	176 223	311	75	57
**A*** - model A without 14 outliers as defined in (Kokh et al. 2018)	80	1 960 441	311	67	44
**Ind** - indazole compounds sub-set of the data-set A	47	1 211 581	311	50	40
**MF** - only molecular descriptors	94	-	-	-	11

Supplementary Table 4.

Microsoft Excel Table containing SMILES for all compounds used in the study (separate file Supplementary Table).

2.2 Supplementary Figures

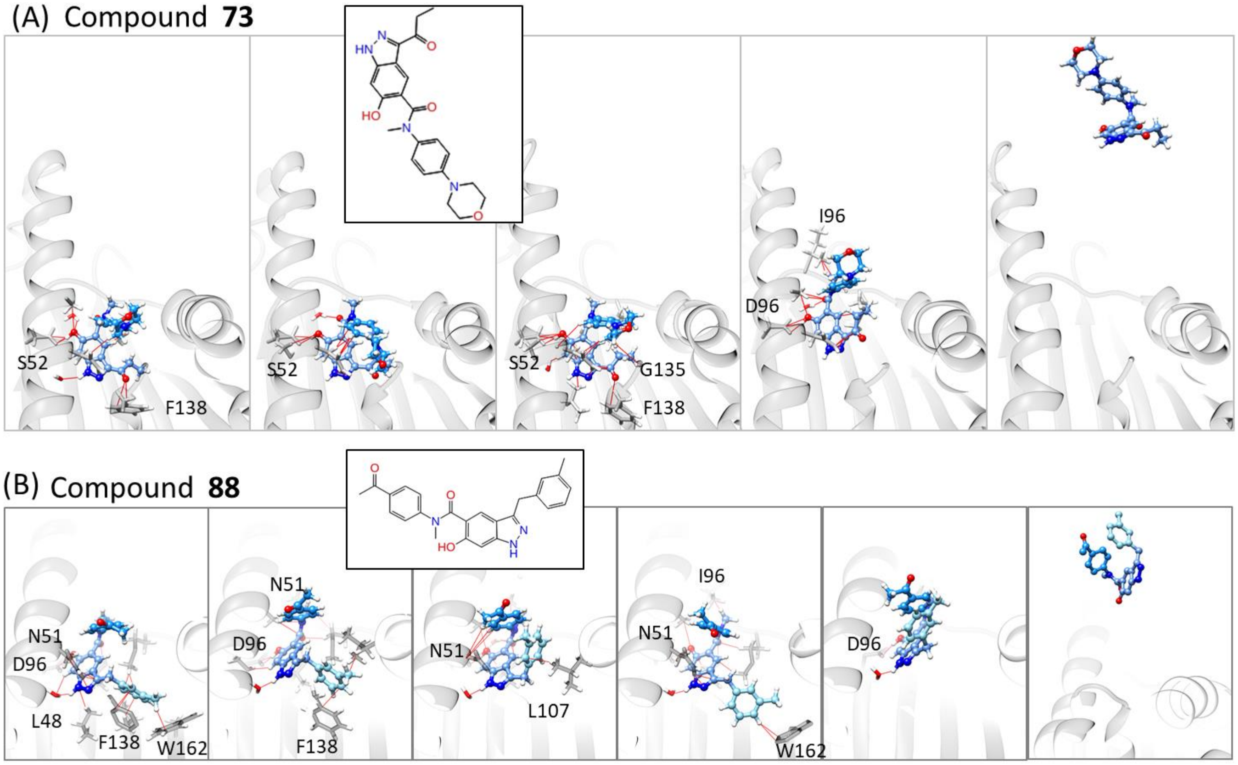

Supplementary Figure 1. Illustration of ligand dissociation from the bound (left) to the unbound state (right) for two indazole compounds, (A) compound **73**, with an exposed 4-(4-morpholinyl) phenyl R_1 fragment, and (B) compound **88**, with a buried 3-methylbenzyl R_2 moiety. Protein-ligand H-bonds and van der Waals contacts are shown by red lines (Images were generated using the Chimera software (Pettersen et al. 2004))

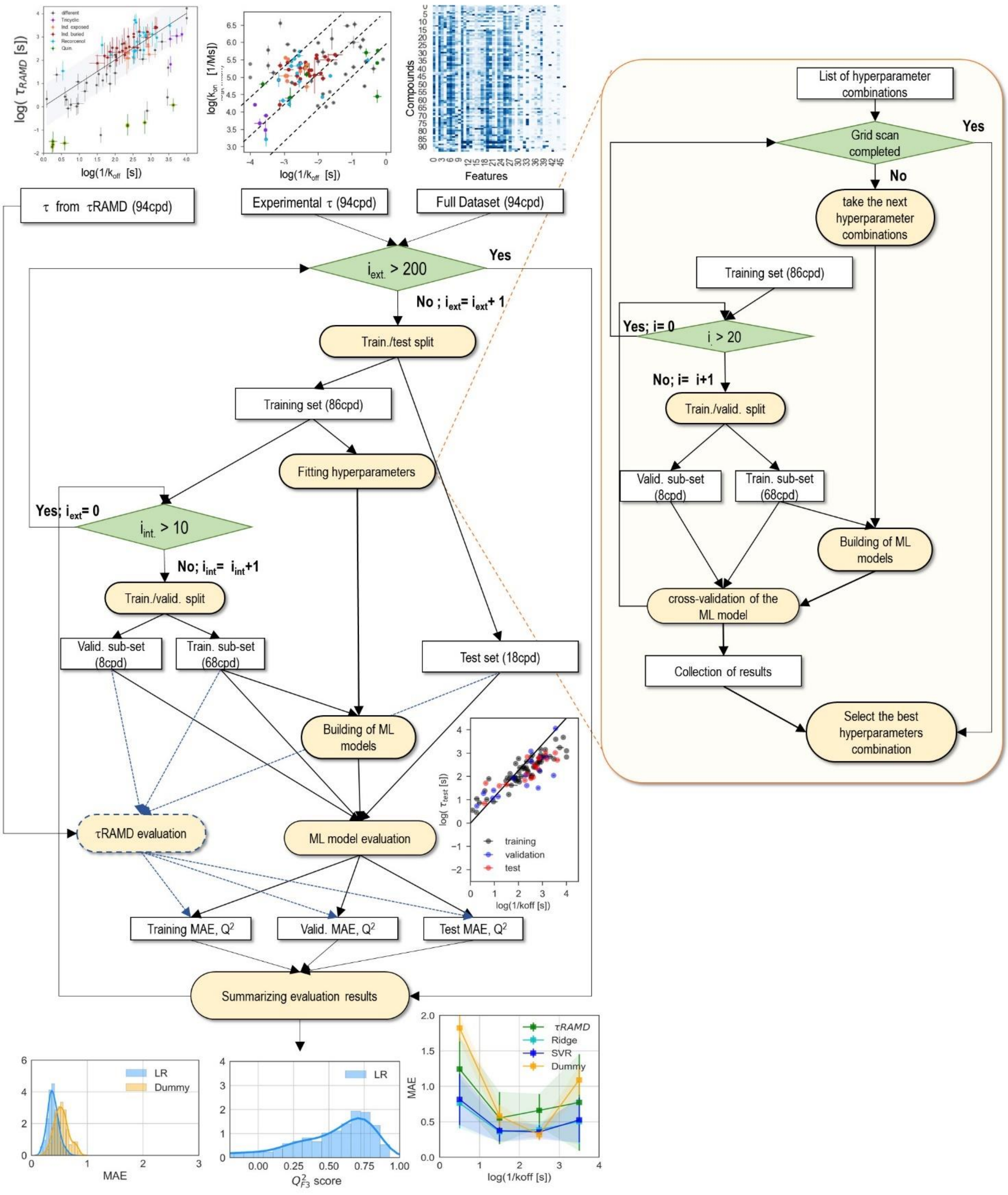

Supplementary Figure 2. Illustration of the building and evaluation of regression models. The workflow was applied to each type of dataset (A, B, C, see main text). The following regression models were employed: linear regression, LR, support vector regression, SVR. Additionally, a Dummy model and the results of τRAMD simulations were evaluated for comparison. Computational

procedures are highlighted in light-orange, while the generated data are shown with a white background; indices i_{int} and i_{ext} define repeats over internal training and external test sets.

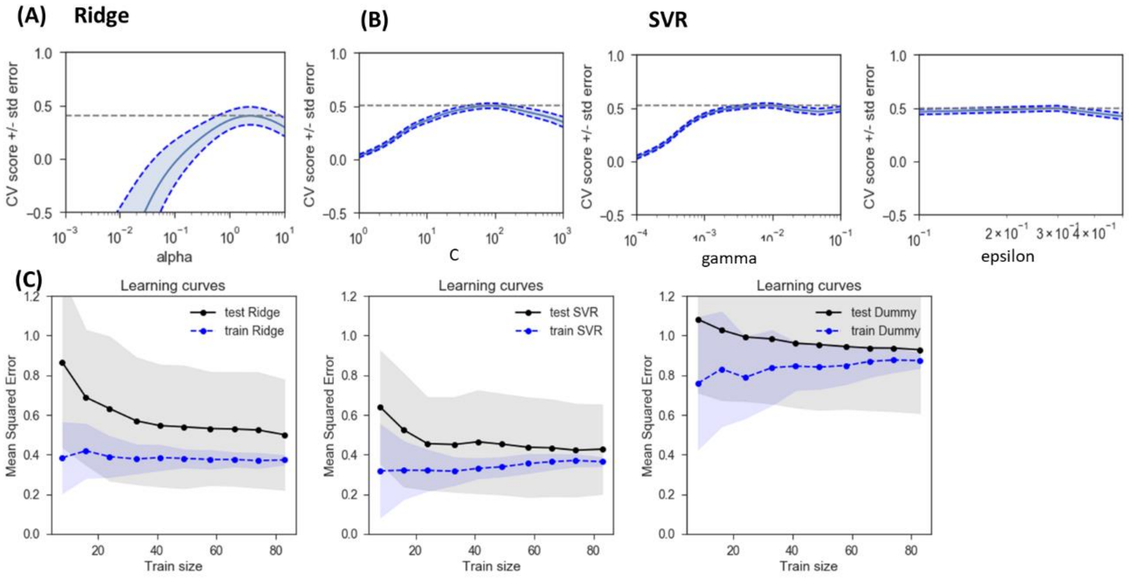

Supplementary Figure 3. Illustration of the fitting of hyperparameters during training. (A) Linear LR model: cross-validation score vs coefficient of the regularization parameter; (B) SVR model: regularization parameter C, kernel coefficient γ, and the regularization width ε; (C) Typical learning curves for the linear LR, SVR, and Dummy models; the standard deviation is shown by the shaded area.

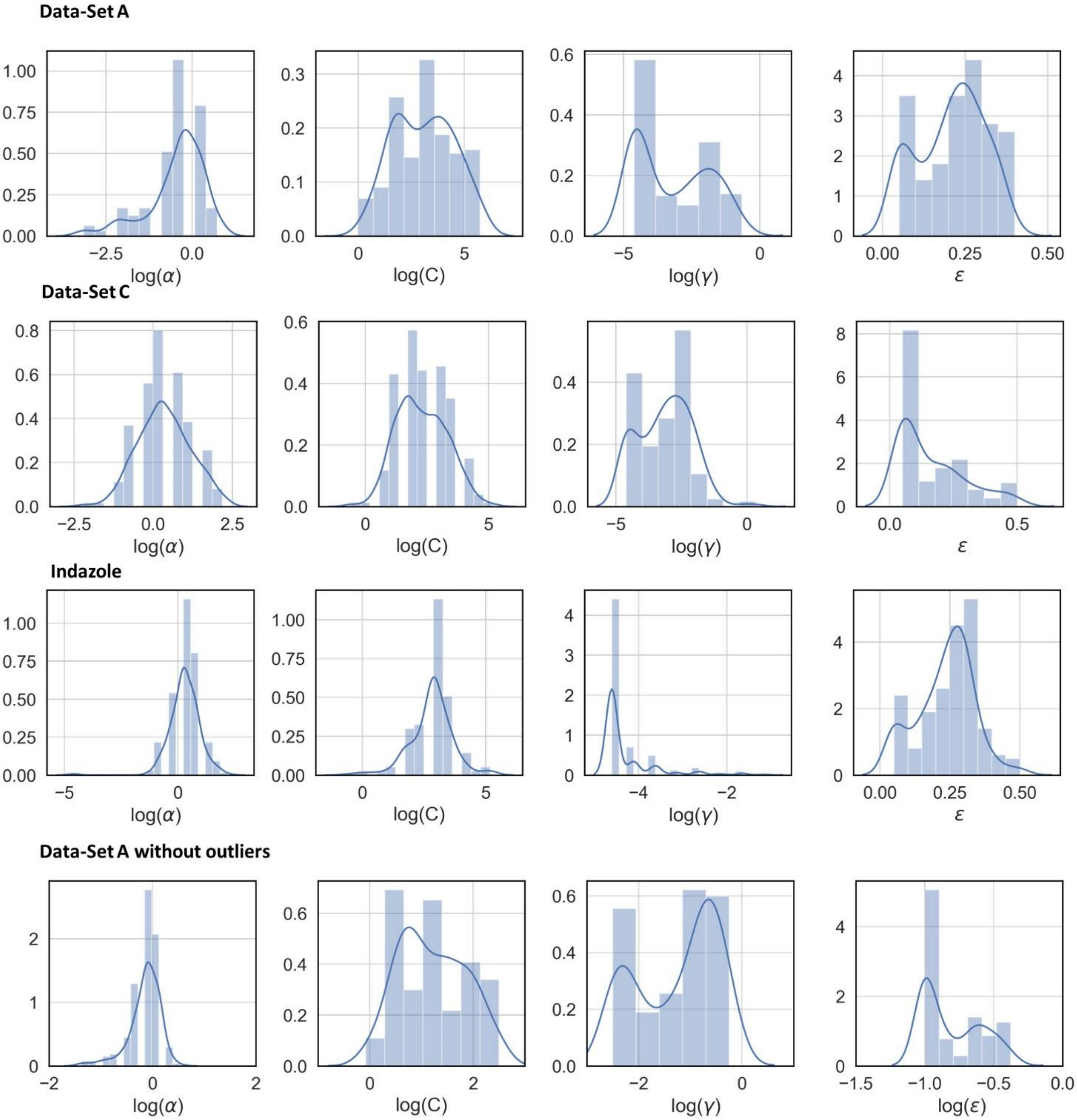

Supplementary Figure 4. Distribution of fitted hyperparameters (histogram and Gaussian kernel density are shown in light-blue bars and blue line, respectively) obtained in 200 training set/test set splits for four different models are shown as histograms: α – regularization parameter in LR; C, γ, ε – parameters of the SVR model as defined in Supplementary Data.

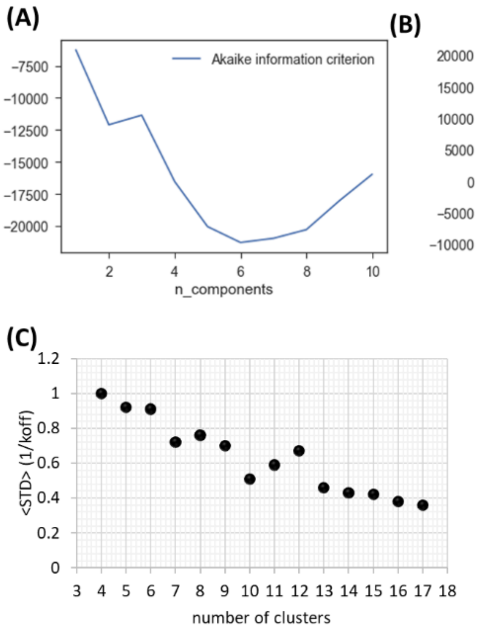

Supplementary Figure 5. (A, B) Variation of Akaike information criterion as a function of the number of clusters is shown for the clustering of (A) the complete data set and (B) only indazole compounds. The optimal number of clusters is at the minimum value of the Akaike information criterion. (C) Variation of the sum of standard deviations of the residence time distribution from the mean in each cluster computed for all clusters as a function of the number of clusters for the complete data set.

Buried fragment

	Ref. Name	Details
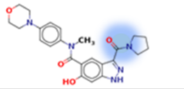	R2:O/ON	Carbonyl oxygen gruppe at the entrance to the hydrophobic sub-pocket
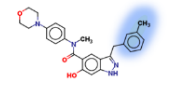	R2:Pm	3-Methylbenzyl
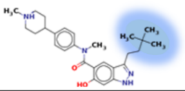	R2:Al	Aliphatic chain
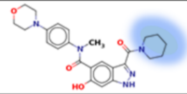	R2:Cy	Cyclic fragment
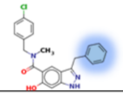	R2:P	Phenyl group
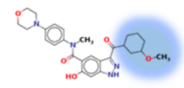	R2:-O	Carbonyl oxygen in a fragment attached to a cyclic group

Solvent exposed fragment

	Ref. Name	Details
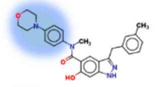	R1:M	4-(4-Morpholinyl)phenyl
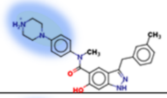	R1:N	Phenylpiperazine
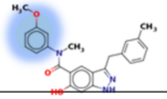	R1:-O	phenyl hydroxyl or carboxyl group
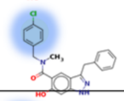	R1:R1: PCl	chlorophenyl
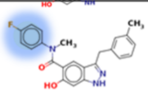	PF	fluorophenyl
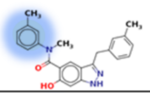	R1:Pm	Methylbenzyl
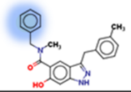	R1:P	Phenyl group

Supplementary Figure 6. Types of molecular fragments used for the analysis of the distribution of indazole compounds over the clusters.

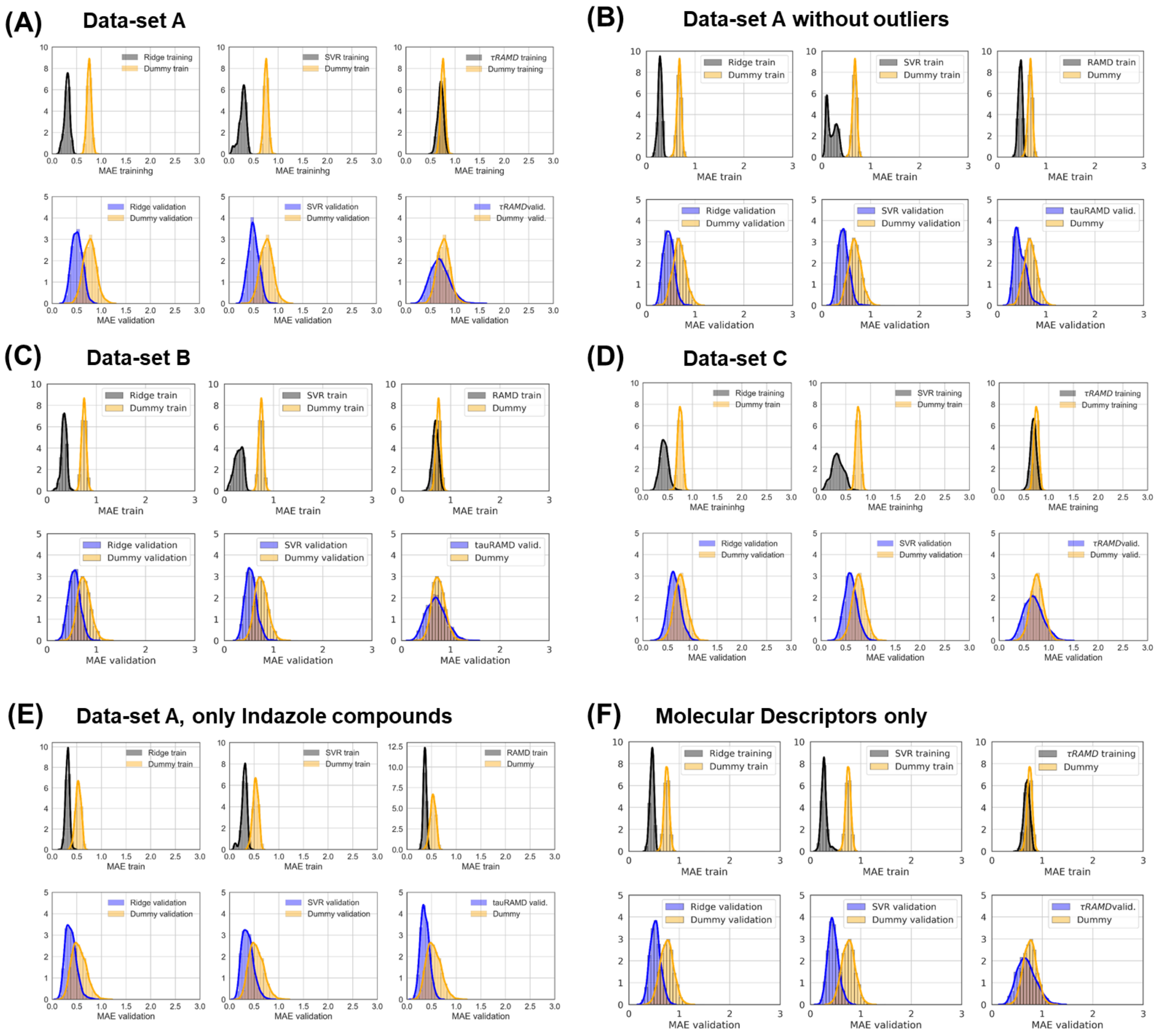

Supplementary Figure 7. Results of RM optimization for data-sets considered in the study. Histograms of MAE for the training and validation sets are shown for two RMs and τRAMD simulations along with that for the Dummy-model.

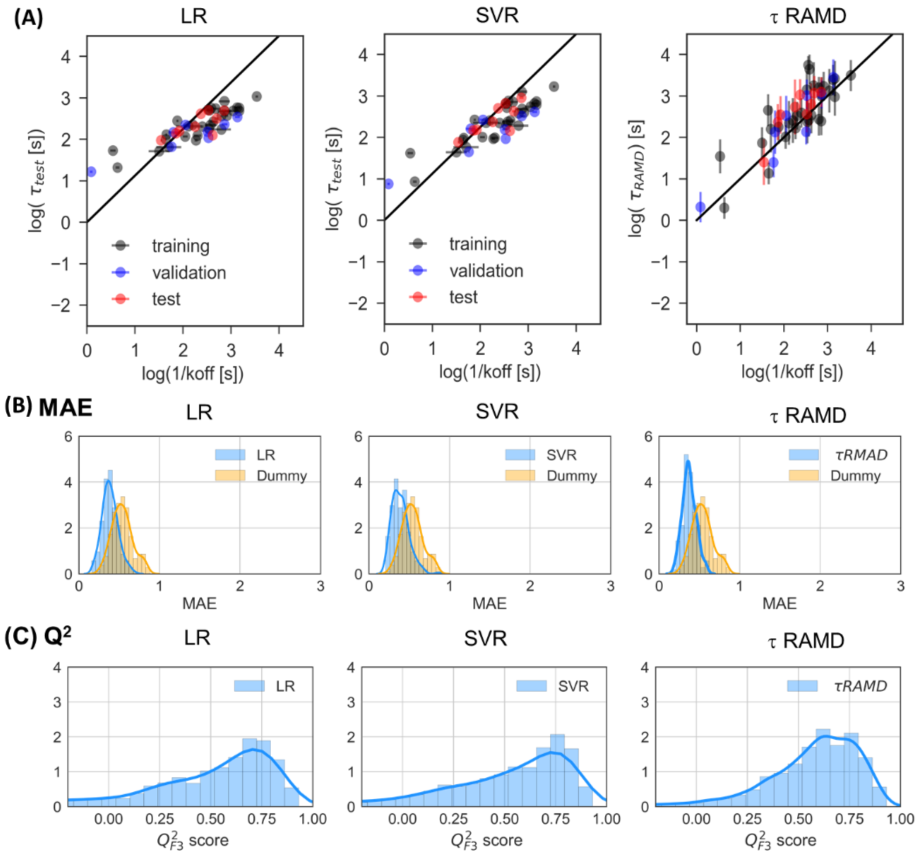

Supplementary Figure 8. Simulation results and model quality assessment for a sub-set of indazole-based compounds of the data-set A: (A) Representative computed vs experimental residence times obtained using linear (LR) and non-linear (SVR) ML models, as well as the τRAMD residence time estimation procedure, black/ blue and red points belong to the training/validation and test sets, respectively. (B-C) Histograms of mean absolute error, MAE (B) and Q^2_{F3} score obtained for 200 repeated training/test splitting for LR, SVR and the τRAMD simulations (blue). Results for the Dummy model are shown in orange.

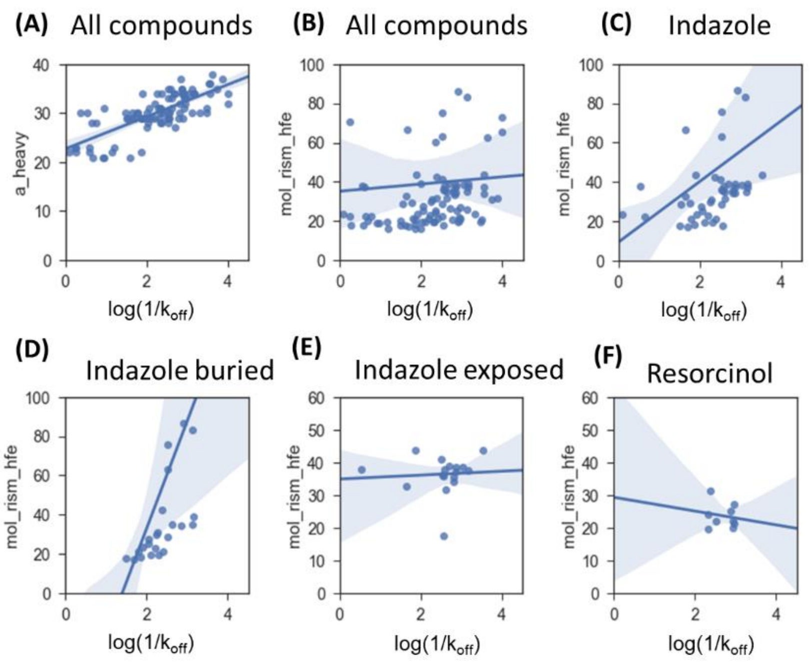

Supplementary Figure 9. Correlation of the number of heavy atoms (A) and desolvation energy (B-F) with measured log(1/k_{off}) values. (A, B) All 94 compounds, R^2 = 0.75 and 0.04, respectively; (C) all indazole compounds, (D, E) indazole compounds with different buried (D) and exposed (E) fragments, (F) – subset of resorcinol compounds (see Fig. 1 D of the main text) . R^2 =0.17, 0.29, 0.26, -0.16, for (C, D, E, F), respectively

References

Kokh, D. et al., 2018. Estimation of Drug-Target Residence Times by τ - Random Acceleration Molecular Dynamics Simulations. *J. Chem. Theory Comput*, 14(7), pp.3859–3869.

MOE, 2017. Molecular Operating Environment (MOE), 2013.08; Chemical Computing Group Inc., 1010 Sherbooke St. West, Suite #910, Montreal, QC, Canada, H3A 2R7.

Pettersen, E.F. et al., 2004. UCSF Chimera--a visualization system for exploratory research and analysis. *Journal of computational chemistry*, 25(13), pp.1605–12.

Schuetz, D.A. et al., 2018. Ligand Desolvation steers on-rate and impacts Drug Residence Time of Heat shock protein 90 (Hsp90) Inhibitors. *Journal of Medicinal Chemistry*, 90(10), pp.4397–4411.

Todeschini, R., Ballabio, D. & Grisoni, F., 2016. Beware of Unreliable Q2! A Comparative Study of Regression Metrics for Predictivity Assessment of QSAR Models. *Journal of Chemical Information and Modeling*, 56(10), pp.1905–1913.
